# Identification and characterization of novel factors that act in the nonsense-mediated mRNA decay pathway in nematodes, flies and mammals

**DOI:** 10.15252/embr.201439183

**Published:** 2014-12-01

**Authors:** Angela Casadio, Dasa Longman, Nele Hug, Laurent Delavaine, Raúl Vallejos Baier, Claudio R Alonso, Javier F Cáceres

**Affiliations:** 1 MRC Human Genetics Unit, Institute of Genetics and Molecular Medicine, Western General Hospital, University of EdinburghEdinburgh, UK; 2 School of Life Sciences, University of SussexBrighton, UK

**Keywords:** *C. elegans*, nonsense-mediated decay, RNAi screen, *smg* genes

## Abstract

Nonsense-mediated mRNA decay (NMD) is a surveillance mechanism that degrades mRNAs harboring premature termination codons (PTCs). We have conducted a genome-wide RNAi screen in *Caenorhabditis elegans* that resulted in the identification of five novel NMD genes that are conserved throughout evolution. Two of their human homologs, *GNL2* (*ngp-1*) and *SEC13* (*npp-20*), are also required for NMD in human cells. We also show that the *C. elegans* gene *noah-2*, which is present in *Drosophila melanogaster* but absent in humans, is an NMD factor in fruit flies. Altogether, these data identify novel NMD factors that are conserved throughout evolution, highlighting the complexity of the NMD pathway and suggesting that yet uncovered novel factors may act to regulate this process.

## Introduction

The NMD pathway targets mRNAs harboring premature termination codons (PTCs) for degradation, but also regulates the stability of a wide array of endogenous transcripts [reviewed by 1–3]. Genetic screens in the nematode *Caenorhabditis elegans* resulted in the identification of seven genes required for NMD, termed *smg-1-7* (for suppressor with morphological effect on genitalia). Likewise, a similar approach in *Saccharomyces cerevisiae* identified three NMD genes, *UPF1-3* (for up-frameshift), that are orthologs of *C. elegans smg-2, smg-3* and *smg-4*, respectively. Subsequently, orthologs for all the *smg* genes were identified in several species including insects, plants and mammals [reviewed by 4,5]. A genome-wide RNAi screen in *C. elegans* identified two additional NMD factors that are conserved throughout evolution and, unlike the core *smg-1-7* genes, are essential for embryonic development 6. Accordingly, they were termed *smgl-1* and *smgl-2* (for *smg-lethal*-*1* and *2*, respectively). Their human homologs, NBAS (for neuroblastoma amplified sequence) and DHX34, act in concert with core NMD factors to co-regulate a large number of endogenous RNA targets 7.

The ATP-dependent RNA helicase, UPF1/SMG2, is a central NMD factor and undergoes cycles of phosphorylation and dephosphorylation that are essential for its activity. UPF1 is phosphorylated at multiple [S/T]Q motifs at its C- and N-terminus by the SMG1 complex, which contains the protein kinase SMG1 and the SMG8,9 subunits 3. NMD is initiated by the assembly of the SURF complex, comprising SMG1, UPF1 and the translation release factors eRF1 and eRF3, in the vicinity of a PTC. Subsequently, an interaction of this complex with an exon junction complex (EJC), deposited downstream as a consequence of the splicing process, leads to the formation of the decay-inducing complex (DECID) that results in mRNA degradation 8. The interaction of the SURF complex with the EJC allows the binding of UPF2 to the N-terminal domain of UPF1 resulting in a large conformational change that activates the UPF1 helicase activity 9,10. UPF1 dephosphorylation is carried out at a later stage and requires the activity of SMG5-7 together with protein phosphatase 2A (PP2A) [reviewed by 11].

In order to establish whether there are more factors that could regulate the NMD pathway within the context of a multicellular organism, we carried out a genome-wide RNAi screen in *C. elegans* that builds on the success of our previous effort 6, but using a different RNAi library that included many previously untested genes. We identified five novel NMD genes that are highly conserved throughout evolution and demonstrate that they participate in the NMD pathway in human cells and *Drosophila* embryos.

## Results and Discussion

### A genome-wide RNAi screen to identify new genes required for NMD

**Figure 1:**
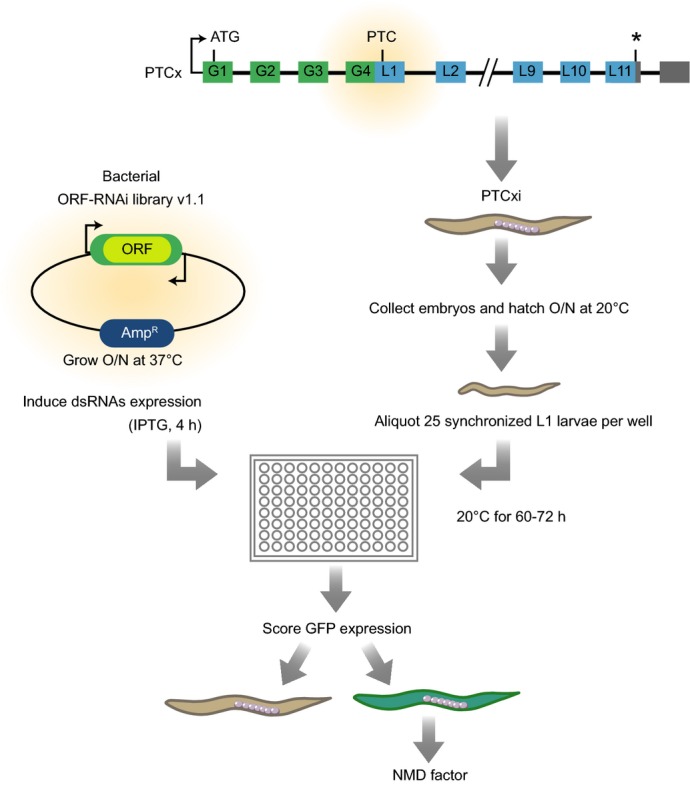
A genome-wide RNAi screen designed to identify novel NMD factors in *C. elegans* The PTCx NMD reporter consists of a fusion between GFP (in green) and LacZ (in blue) genes; a PTC is present downstream of the GFP coding region, making the transcript an NMD substrate.

**Figure 2:**
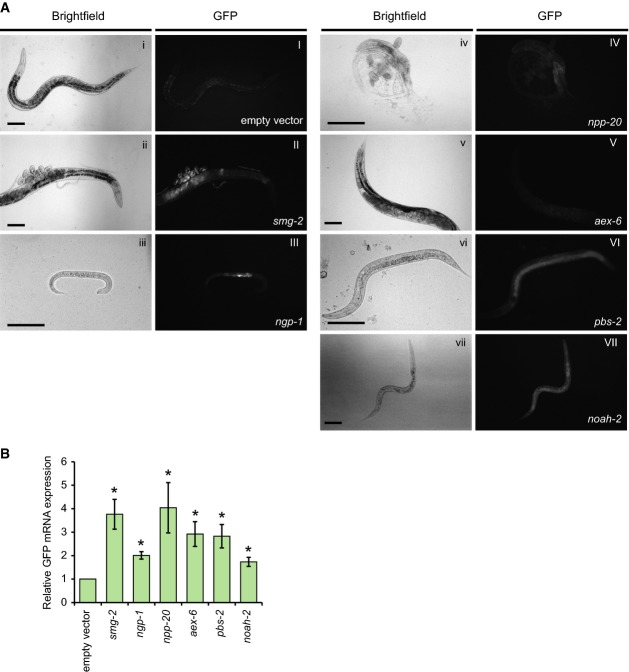
Newly identified NMD factors RNAi was induced with an empty vector as a negative control (panel I), whereas a *smg-2* clone (panel II) was used as a positive control. Panels i and ii show brightfield images of the PTCxi strain treated with the negative and positive controls, respectively. Depletion of five genes (panels III to VII) resulted in increased GFP expression. Panels iii to vii show brightfield images of the phenotypes of the affected worms. The scale bars correspond to 100 μm.Depletion of the novel NMD genes in *C. elegans* leads to upregulation of the PTCx NMD reporter mRNA, which was monitored by quantitative RT–PCR relative to the expression of *ama-1* reference gene. The values shown are the average fold-change (mean ± SEM) from at least three independent experiments relative to empty vector-depleted worms. Statistical analysis was performed using the Mann–Whitney *U*-test for non-parametric distributions. *P <* *0.05.

Our previous RNAi screen in *C. elegans* led to the identification of *smgl-1*/*NBAS* and *smgl-2*/*DHX34* that act in NMD in nematodes and vertebrates 6,12. We revisited this approach with the use of a different RNAi library: the *C. elegans* ORF-RNAi library v.1.1 that contains 11,511 clones targeting 55% of the nematode genome 13. This library includes dsRNAs against 1,736 genes that were not targeted previously 14. As earlier, we used the *C. elegans* PTCxi strain that expresses a GFP-based reporter harboring a PTC that is integrated in the genome 6 (Fig1). This PTCx reporter has reduced GFP expression, since its transcript is subject to NMD-mediated degradation. Thus, novel NMD genes were identified by the criterion that their silencing by RNAi restores GFP expression. Accordingly, we searched for the appearance of green worms, dead or alive, following inactivation of individual genes by RNAi. As a negative control, we fed PTCxi animals empty RNAi vector, which had no effect on the level of GFP expression (Fig2A, panel I), whereas inactivation of the core NMD factor, SMG-2/UPF1, which induced strong GFP expression, was used as a positive control (Fig2A, panel II). Screening of the entire library resulted in the identification of five RNAi clones that scored positive by increased GFP expression (Fig2A*,* panels III–VII). The clones identified in this screen are: T19A6.2, Y77E11A.13, Y87G2A.4, C47B2.4 and F52B11.3 (Table1). Confirming that these newly identified *C. elegans* genes act as NMD factors, quantitative RT–PCR analysis showed that downregulation of each of these genes led to an increase in the GFP reporter mRNA level, as was seen with depletion of *smg-2* (Fig2B).

**Table 1:** List of novel putative NMD factors identified in this study

Clone ID	Gene name (*C. elegans*)	Predicted function in *C. elegans*	*C. elegans* phenotype	Gene name (*H. sapiens*)
T19A6.2	*ngp-1*	Nuclear/nucleolar GTP-binding protein family	Embryonic lethalLarval arrestMaternal sterile	*GNL2*
Y77E11A.13	*npp-20*	Nuclear pore complex protein	Embryonic lethalLarval arrest	*SEC13*
Y87G2A.4	*aex-6*	Rab protein involved in trafficking of vesicles	Aboc expulsion missingConstipated	*RAB27ARAB27B*
C47B2.4	*pbs-2*	Proteasome p subunit	Embryonic lethalLarval arrest	*PSMB7PSMB10*
F52B11.3	*noah-2*	PAN and ZP domain-containing protein	Embryonic lethalLarval arrest	Not conservedConserved in *Drosophila* (*nompA*)

### Novel NMD genes in *C. elegans*

All of the newly identified genes, with the exception of *noah-2*, are conserved throughout evolution and have clear orthologs in human, mouse, zebrafish and yeast ( Supplementary Figs S1 and S2). The *C. elegans* gene *ngp-1* (T19A6.2) corresponds to the human *GNL2* gene and encodes a putative GTPase that comprises a GTP-binding domain formed by five G-motifs, which is typical of the HSR1_MMR1 GTP-binding protein subfamily. It also contains a conserved N-terminal domain (NGP1NT) (Supplementary Fig S1A). Its yeast homolog, Nog2p, is involved in ribosomal biogenesis playing a role in the processing of the pre-60S particles 15. The *npp-20* gene (Y77E11A.13) corresponds to human *SEC13*, which encodes a protein that comprises six WD-40 domains (Supplementary Fig S1B) and is a constituent of the endoplasmic reticulum and the nuclear pore complex (NPC) 16. The *aex-6* gene (Y87G2A.4) is a member of the Rab small GTPase superfamily. It has two homologs in humans, RAB27A and RAB27B (Supplementary Fig S1C), with RAB27B functioning in the trafficking of dense-core vesicles 17. The *pbs-2* gene (C47B2.4) is a member of the proteasome B-type family and is a 20S core beta subunit of the proteasome (Supplementary Fig S1D), with two human homologs, *PSMB7* and *PSMB10* 18. Finally, the *noah-2* gene (F52B11.3) encodes a PAN and ZP domain-containing protein that is required for embryonic and larval development, reproduction, coordinated locomotion and molting (Supplementary Fig S1E) 19. It is related to the *Drosophila* extracellular matrix component *nompA* (no-mechanoreceptor-potential A) 20. There are no homologs of *noah-2* in vertebrates (Supplementary Fig S2).

### The newly identified NMD genes are required for proper development in *C. elegans*

Depletion of all these novel genes resulted in developmental defects, in contrast to *smg-2* depletion that did not compromise development (Fig2A, compare panels ii with iii–vii). Thus, these novel NMD factors are different from core *smg-1-7* genes and display similar behavior to *smgl-1, 2* that are essential for viability 6. In *C. elegans*,*ngp-1* is an essential gene. Its knockdown led to a variable larval arrest, where the majority of the affected worms were arrested at L1–L2 stages, compared to control worms that invariably reached adulthood within the time limit of the experiment (Fig2A, panels i and iii, respectively). Those worms that escaped early arrest failed to reach adulthood and produced no embryos. Depletion of *npp-20* resulted in worms arrested at L2–L3 larval stages. The majority of the worms were very fragile and died by bursting (Fig2A, panel iv). By contrast, depletion of *aex-6* resulted in a mild but highly consistent phenotype with worms able to progress through the developmental stages normally; however, adult worms were constipated, as previously reported 21, and also exhibited an egg-laying defect and reduced brood size (Fig2A, panel v). Depletion of *pbs-2* resulted in very sick and pale larvae that were arrested around L2 stage, displaying a swollen intestine in the majority of the affected worms (Fig2A, panel vi). Finally, depletion of *noah-2* led to an early larval arrest at L2–L3 stages and subsequent larval lethality (Fig2A, panel vii).

### *Drosophila nompA* gene is required for NMD

**Figure 3:**
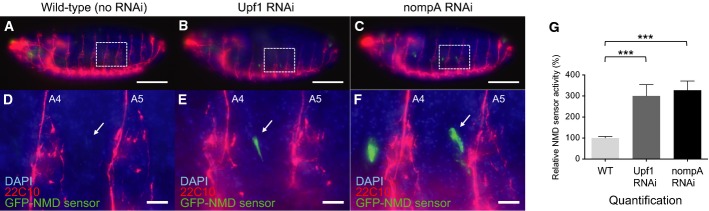
*Drosophila nompA* is required for NMD in *Drosophila* embryos A–C Embryos expressing a GFP-NMD sensor (green) under the control of *nompA* regulatory sequences show signal in support cells linked to the embryonic peripheral nervous system (PNS) [labeled by 22C10 signal (magenta)]. DAPI signal is shown in blue. Expression of UAS-RNAi constructs against *Upf1* (B) or *nompA* (C) genes using a *nompA*-Gal4 driver leads to upregulation of the GFP-NMD sensor when compared to wild-type (no RNAi) (A), revealing a reduction in NMD activity in the knockdown conditions. Scale bars represent 100 μm.D–F Higher magnification (40×) of the areas marked by a rectangle in (A–C) further illustrates the upregulation of the GFP-NMD sensor in Upf1- (E) and NompA-depleted cells (F). Optical fields include embryonic abdominal segments A4–A5. Scale bars represent 10 μm.G Quantification of GFP signal in cells marked by an arrow in panels (D–F) shows a significant upregulation of NMD sensor expression upon downregulation of Upf1 (dark gray) and NompA (black) compared to wild-type (no RNAi) (light gray). Results represent the average of five biological replicates (mean ± SEM). Pair-wise comparisons were performed using a one-tailed *t*-test (non-parametric) between treatments and wild-type. ***P <* *0.001.

There is no ortholog of *noah-2* in mammalian genomes; nonetheless, the gene is clearly present in *Drosophila melanogaster* (Supplementary Fig S2), suggesting that it most likely emerged at some point during the early evolution of the Ecdyzozoa before the split between arthropods and nematodes. We assessed its potential role in NMD in *Drosophila* embryos by means of a previously described NMD fluorescent GFP reporter 22,23. Unlike the previously characterized *Drosophila* NMD genes *upf1*,*upf2* and *upf3* that are ubiquitously expressed 22,24, expression of *nompA* in *Drosophila* embryos is confined to type I sense organs of the peripheral nervous system (PNS) 20. Thus, we used the UAS/Gal4 system 25 to drive expression of a *nompA* RNAi construct within the PNS using a NompA-Gal4 line 26. We found that *nompA* knockdown within its expression domain in the embryonic PNS led to a significant upregulation of the NMD reporter (Fig3C, F and G), which was comparable to the effect observed following UPF1 depletion (Fig3B, E and G), as compared to no RNAi treatment (Fig 3A, B and D), which showed no expression of the reporter. This demonstrates that the *Drosophila* ortholog of *noah-2* behaves as a tissue-specific NMD factor in fruit fly embryos.

### GNL2 and SEC13 act in the NMD pathway in human cells

**Figure 4:**
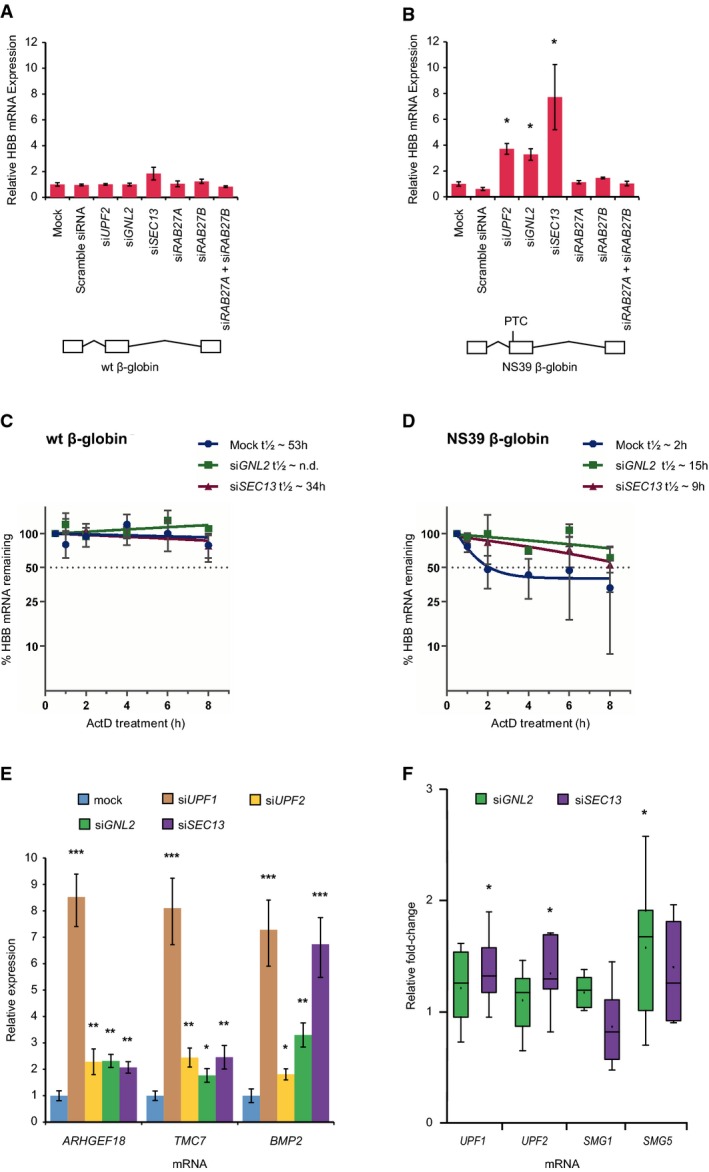
GNL2 and SEC13 are required for NMD in human cells A, B HeLa cells stably expressing a wild-type β-globin reporter (A) or a β-globin NS39 NMD reporter (B) were mock-depleted or depleted of UPF2, GNL2, SEC13, RAB27A, RAB27B or both RAB27A and RAB27B. The level of the β-globin mRNA was monitored by quantitative RT–PCR relative to two reference genes (*POLR2J* and *ACTB*). The values shown are the average fold-change (mean ± SEM) from four independent experiments relative to mock-depleted cells (control). Statistical analysis was performed using the Mann–Whitney *U*-test for non-parametric distributions. *P <* *0.05. The level of depletion of NMD factors is shown in Supplementary Fig S3C.C, D Analysis of the half-life of β-globin reporters. Samples were collected at the indicated time points, and the mRNA levels of the *HBB* reporters were monitored by qRT–PCR and normalized to *POLR2J* and *ACTB* reference genes. The values shown are the average fold-change (mean ± SEM) from three independent experiments relative to the first time point.E Depletion of GNL2 and SEC13 leads to a significant upregulation in the mRNA levels of endogenous NMD substrates. Samples were analyzed as described in (A, B) *P <* *0.05; ***P <* *0.01, ***P <* *0.001.F GNL2 and SEC13 contribute to the negative NMD feedback loop, regulating the levels of transcripts encoding NMD factors. RT–qPCR analysis of total cellular RNA from HeLa cells depleted of GNL2 (in green) and SEC13 (in purple) is shown. The graph shows distribution of relative fold-change from eight independent experiments relative to mock-depleted cells (control). Statistical analysis was performed using Student's *t*-test. *P <* *0.05.

Next, we investigated a potential role for the human homologs of the factors identified in this screen in NMD in human cells. HeLa cells stably expressing an integrated human β-globin (*HBB*) gene, either in a wild-type version or carrying an NMD-inducing mutation (NS39) 27, were individually depleted of each of these genes. The level of depletion of these factors is shown in Supplementary Fig S3C. As expected, depletion of the human homologs of the novel NMD factors did not significantly affect the levels of the wild-type β-globin mRNA (Fig4A). By contrast, depletion of UPF2 (positive control) or of GNL2 and SEC13, but not of RAB27A-B (depleted individually or in combination), resulted in a significantly increased level of the β-globin NMD reporter (NS39) mRNA when compared to mock-depleted cells (Fig4B). Whereas individual depletion of PSMB10 clearly showed no effect on the levels of the NMD reporter, knockdown of PSMB7 (either individually or in combination with PSMB10) led to an upregulation of both the wild-type and NMD reporters, making it difficult to conclude whether PSMB7 had a specific role in NMD (Supplementary Fig S3A and B). Altogether, these experiments show that *GNL2* and *SEC13* have a clear effect in the NMD response in human cells, whereas it still remains possible that the remaining tested genes may have an NMD effect that is substrate or tissue specific.

To rule out indirect effects of GNL2 and SEC13, we first investigated whether these factors have a general role in mRNA translation, which would impact on NMD. This is unlikely, since the very nature of the RNAi screen in *C. elegans* requires that the NMD reporter is indeed translated. In agreement, knockdown of GNL2 or SEC13 did not result in a general inhibition of translation, as measured by metabolic labeling of HeLa cells (Supplementary Fig S3E and F). Next, we examined the half-life of the wild-type or NMD-sensitive NS39 β-globin reporter mRNAs upon depletion of GNL2 and SEC13. The stability of wild-type β-globin mRNA was unaffected by GNL2 or SEC13 depletion (Fig4C). By contrast, depletion of GNL2 or SEC13 led to a marked stabilization of a PTC-containing β-globin mRNA, confirming that both GNL2 and SEC13 act in the NMD pathway (Fig4D). Furthermore, depletion of GNL2 or SEC13 in HeLa cells led to a marked upregulation of three endogenous transcripts that were previously reported to be sensitive to NMD regulation 7,28 (Fig4E). In agreement, knockdown of GNL2 or SEC13 also resulted in an increased half-life of one of those NMD substrates (*ARHGEF18*) mRNA (Supplementary Fig S3D). As further proof of the role of the novel factors identified in this screen in the NMD pathway, we probed for the interaction of GNL2 with the core NMD factor UPF1. We immunopurified Flag-tagged UPF1 expressed at physiological levels from transiently transfected HEK 293T cells that also co-expressed T7-tagged GNL2 in the presence of RNase A. We used transiently expressed Flag-empty vector (F-EV) co-expressed with T7-tagged GNL2, as a negative control. We observed that UPF1 specifically co-immunoprecipitated with T7-tagged GNL2 in an RNA-independent manner (Supplementary Fig S4). Future studies will aim to test the interaction of GNL2 and SEC13 with components of the NMD machinery.

### GNL2 and SEC13 participate in an autoregulatory feedback loop

Transcripts encoding NMD factors are sensitive to depletion of different NMD factors as part of a negative feedback regulatory loop that acts to tightly control NMD homeostasis 29,30. We investigated whether depletion of GNL2 or SEC13 would have an impact on the levels of transcripts encoding NMD factors in human cells. Interestingly, we found that depletion of GNL2 in HeLa cells resulted in a significant upregulation of the levels of *SMG5* mRNA, as well as increased mRNA levels for *UPF1*,*UPF2* and *SMG1* (Fig4F). Similarly, SEC13 depletion resulted in a significant upregulation in the levels of mRNAs encoding UPF1 and UPF2, and to a lesser extent of *SMG5* mRNA (Fig4F). Thus, the novel NMD factors, GNL2 and SEC13, participate in a negative regulatory feedback loop controlling the expression of NMD factor mRNAs.

## Conclusions

Even though the NMD pathway is a highly conserved process, several mechanisms have evolved to define a PTC across different species 3. Whereas in mammalian cells, NMD is linked to pre-mRNA splicing, exon boundaries are not used to define PTCs in other organisms, including *S. cerevisiae* 31, *S. pombe* 32,33, *Drosophila* 34 and *C. elegans* 6.

RNAi screens have been widely used in *C. elegans* to identify genes involved in many different cellular pathways, and we had used this approach in the past to identify novel NMD factors 6. Here, we revisited this approach with the use of a different RNAi library that includes dsRNAs against 1,736 genes that were not targeted in our previous screen and have identified five novel NMD genes that are required for proper development in nematodes. Due to the high degree of evolutionary conservation, we could analyze the role of these newly identified NMD factors in mammalian cells and we chose HeLa cells as our experimental system. The only exception was the *noah-2* gene that does not have a human counterpart. For this, we studied its functional homolog in *Drosophila* and found that it acts in the NMD pathway in insects. Importantly, we show that two human homologs, *GNL2* (*ngp-1*) and *SEC13* (*npp-20*), are also required for NMD in human cells. Only recently, we uncovered the mechanism by which the RNA helicase DHX34, which was identified in our first RNAi screen, promotes mRNP remodeling and triggers the conversion from the SURF complex to the DECID complex resulting in NMD activation 35. Further studies will help to delineate the mechanism by which SEC13 and GNL2 activate NMD in human cells, as well as their involvement in the described alternative NMD branches 36,37,38. In summary, our work has led to the identification of novel NMD factors in nematodes, flies and mammals, revealing that the machinery underlying NMD is more complex than previously thought.

## Materials and Methods

### Genome-wide RNAi screen

The NMD reporter is based on the *GFP::lacZ* vector pDP96.04 and is driven by the ubiquitous *sec-23* promoter 6. The PTCxi transgenic strain carrying this GFP-based NMD reporter integrated in the genome was described previously 6. PTCxi transgenic worms were grown on standard NGM plates seeded with OP50 *E. coli* bacteria at 20°C. The RNAi library used for the screen was created in the laboratory of Marc Vidal and is commercially available 13. RNAi was performed in liquid format by feeding synchronized population of PTCxi L1 larvae with bacterial clones expressing dsRNA corresponding to individual genes in 96-well plates 6. Worms were then scored for the appearance of GFP expression, indicating that the depleted protein is required for NMD in *C. elegans*.

### Fly stocks

We used the following fly stocks all obtained from the Bloomington Stock Center (Indiana, USA): *w*^*1118*^*; P{GMR29A10-GAL4}attP2*,*y*^*1*^ w^*^; P{UAS-mCD8::GFP.L}LL5 (BM 5137), *y^1^ v^1^; P*{*TRiP.JF02919*}*attP2* and *y*^*1*^ *v*^*1*^*; P{TRiP.GL01485}attP2*. Animals were reared at 25°C on cornmeal, molasses and yeast medium.

For more detailed Materials and Methods see the Supplementary Information.

## Supporting Information

Supplementary Information

Review Process File
