# Supplementary figures and images for: Identification and characterization of novel factors that act in the nonsense-mediated mRNA decay pathway in nematodes, flies and mammals
Source: EMBO Rep. 2014 Dec 1;16(1):71–8. doi: 10.15252/embr.201439183 (PMC4304730; doi:10.15252/embr.201439183)

**Figure S1**

**A**

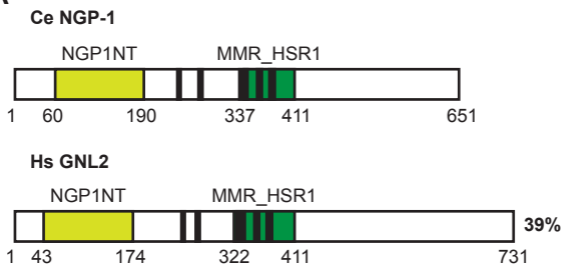

**B**

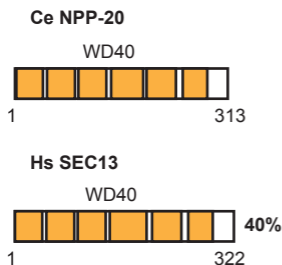

**C**

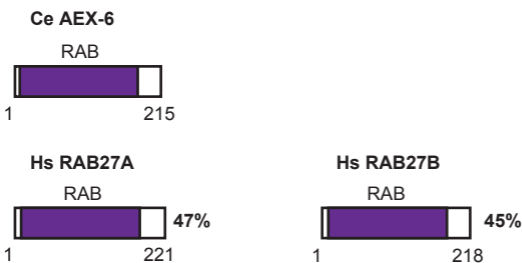

**D**

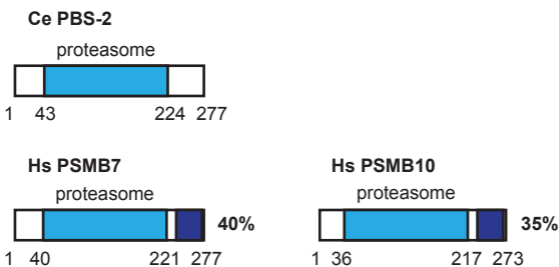

**E**

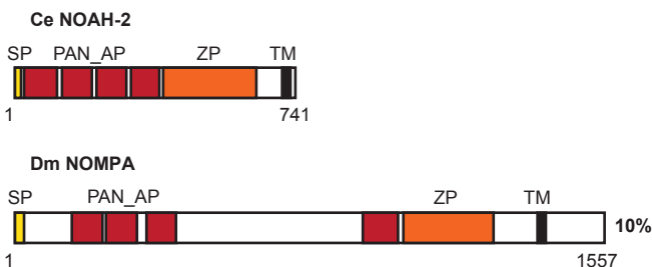

Supplement: Supplementary file 1 [file embr0016-0071-sd1.pdf]

Figure S3

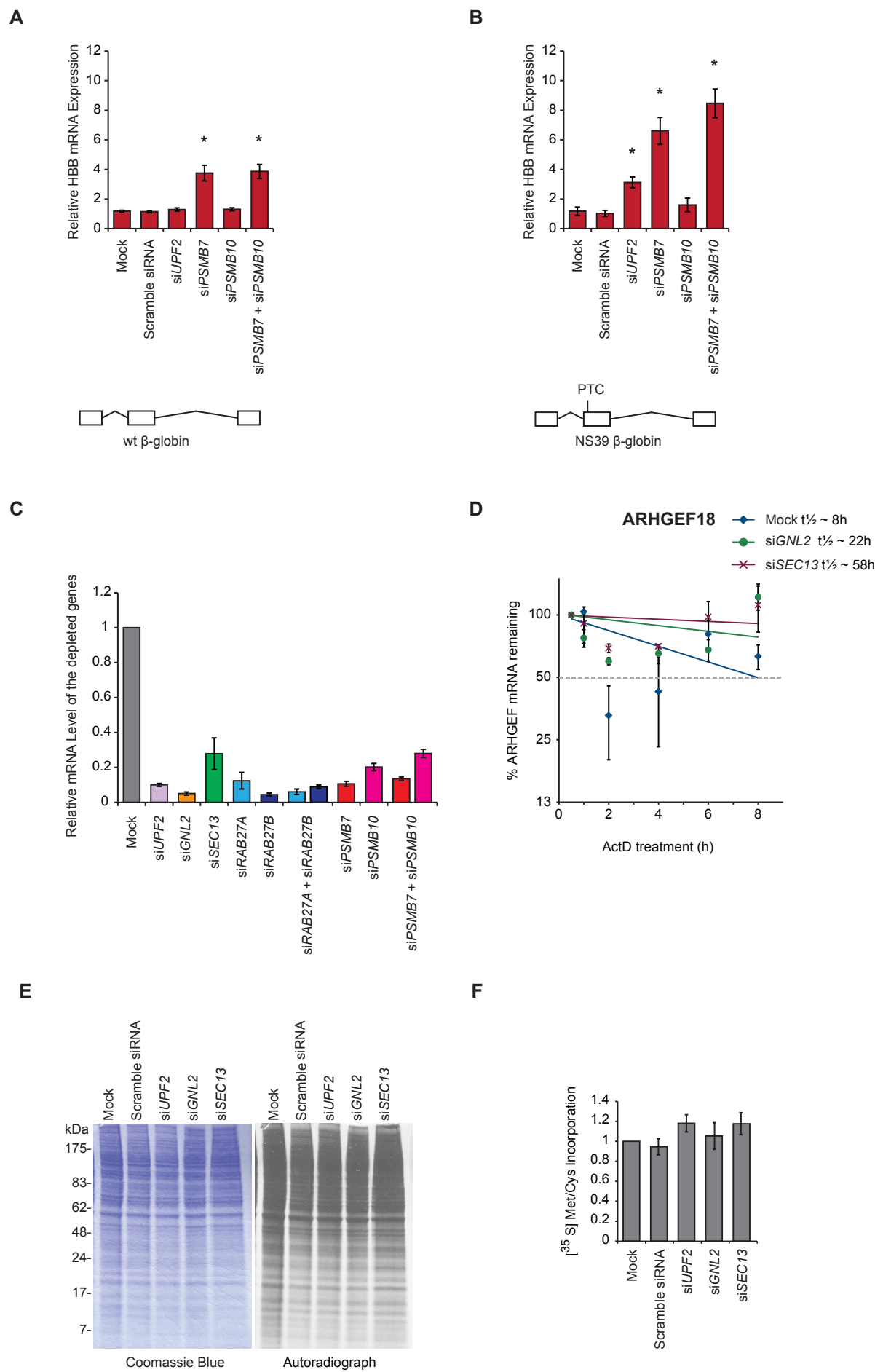

Supplement: Supplementary file 3 [file embr0016-0071-sd3.pdf]

# Figure S4

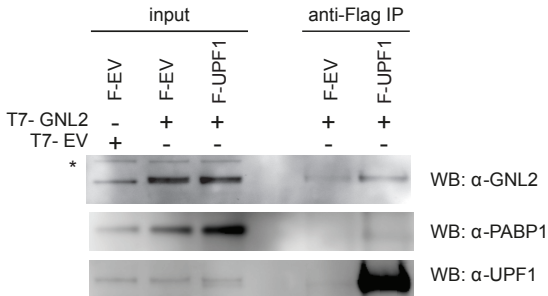

Supplement: Supplementary file 4 [file embr0016-0071-sd4.pdf]
