# Supplementary material for: Identification and characterization of novel factors that act in the nonsense-mediated mRNA decay pathway in nematodes, flies and mammals
Source: EMBO Rep. 2014 Dec 1;16(1):71–8. doi: 10.15252/embr.201439183 (PMC4304730; doi:10.15252/embr.201439183)
Supplement: Supplementary file 2 [file embr0016-0071-sd2.pdf]

Figure S2

A

```
                           *        20         *        40         *        60         *        80         *       100
Hs GNL2    : MVKPKYK-GRS----------------TINPSKASTNPDRVQG--AGGQNMRDRATIRRLNMYR-QKERRNSRGKIIKPLQYQSTVASGTVARVEPNIKW :  80
Mm Gnl2    : MVKPKYK-GRS----------------TINRSAASTNPDRVQG--AGGQNMRDRGTIRRLNMYR-QKERRNSRGKVIKPLQYQSTVASGTVARVEPNIKW :  80
Dr gnl2    : MVKAKFK-GKS----------------SINTSNSSSNPDRVKG--AGGNNMRDRATIKRLNMYR-QKQRCNSRGKVIKPLQYQNTVAPGTVARVEPNIKW :  80
Dm ns2     : MPKVRSTPGKPRTQ-------------GFNHSNHSMNPERPKSGLKGVAHPRTKGTIKRLQMYRNFKAKRDRTGKILTPAPFQGRLPAGTMARVEPTPKW :  87
Sc NOG2    : MGTGKKEKSRR-----------------IREGDTKDGNLRVKG----ENFYRDSKRVKFLNMYTSGKEIRNKKGNLIRAASFQDSTIP--DARVQPDRRW :  77
Ce ngp-1   : MVKARKEGKVSKKLKQKPDYGPVKNRHTFRGSNHSMNPDRKPD--AKDKSQRSKATINRLRMYKSFKPIRDSKGKILKAAPFQDTLASGTQARIEPNRKW :  98

                           *       120         *       140         *       160         *       180         *       200
Hs GNL2    : FGNTRVIKQSSLQKFQEEMDTVMKDPYKVVMKQSKLPMSLLHDRIRPHNLKVHILDTESFETTFGPKSQRKRPNLFASDMQSLIENAEMSTESYDQGKDR : 180
Mm Gnl2    : FGNTRVIKQASLQKFQEEMDKVMKDPYKVVMKQSKLPMSLLHDRIQPHNAKVHILDTESFESTFGPKSQRKRPNLFASDMQSLLENAEMSTESYDQGKDR : 180
Dr gnl2    : FANTKVIKQSSLQKFQEEMNAVKKDPYRVVMRQSKLPMSLLHDRIKAHNSKVHILDTETFETTFGPKAQRKRPNLSVGELKEFAEQAEVSAQSYSAEKDR : 180
Dm ns2     : FSNSRVISQTALQKFQDEIGKAVKDPYQVIMKPSQLPVTLLNEAAKYK--RVHLLDTESFDSTFGPKKQRKRVSLKVRDLEDLSKAADDQADKYDSAKDL : 185
Sc NOG2    : FGNTRVISQDALQHFRSALGETQKDTYQVLLRRNKLPMSLLEEKDADESPKARILDTESYADAFGPKAQRKRPRLAASNLEDLVKATNEDITKYEEKQVL : 177
Ce ngp-1   : FGNTRIIGQEQLQKFQANLGKVLSDPFQVVMKQTKLPISLLQEKAKTQ--RVHVTETESFEYTFGKKSLRKKAKLTEASLEEMSKSAEDRDVKYKPELDL : 196

                           *       220         *       240         *       260         *       280         *       300
Hs GNL2    : DLVT--------EDTGVRNEAQEEIYKKGQSKRIWGELYKVIDSSDVVVQVLDARDPMGTRSPHIETYLKKEKPWKHLIFVLNKCDLVPTWATKRWVAVL : 272
Mm Gnl2    : DLVM--------EDTGVRNEAQEEIYKKGQSKRIWGELYKVIDSSDVVVQVLDARDPMGTRSPHIEAYLKKEKPWKHLIFVLNKCDLVPTWATKRWVAVL : 272
Dr gnl2    : DLVS--------EDSGVKEEAREEIFKKGQSKRIWGELYKVIDSSDVIIQVLDARDPMGTRSQSIETYLKKEKPWKHLIFVLNKCDLIPTWVTKHWVAVL : 272
Dm ns2     : DLIR--------EDTGEKKAVRDWVFGAGQSKRIWNELHKVVDASDVLLQVLDARDPMGTRSKYIEEFLRKEKPHKHLFFILNKVDLVPVWVTQRWVAIL : 277
Sc NOG2    : DATLGLMGNQEDKENGWTSAAKEAIFSKGQSKRIWNELYKVIDSSDVVIHVLDARDPLGTRCKSVEEYMKKETPHKHLIYVLNKCDLVPTWVAAAWVKHL : 277
Ce ngp-1   : SREK---------ELGDRPENMNPLFRAGQSNRVWGELYKVIDSSDVVVQVVDARDPMGTRCRHVEEFLRKEKPHKHLVTVINKVDLVPTWVTRKWIGEL : 287

                           *       320         *       340         *       360         *       380         *       400
Hs GNL2    : SQDYPTLAFHASLTNPFGKGAFIQLLRQFGKLHTDKKQISVGFIGYPNVGKSSVINTLRSKKVCNVAPIAGETKVWQYITLMRRIFLIDCPGVVYPSE-D : 371
Mm Gnl2    : SQDYPTLAFHASLTNPFGKGAFIQLLRQFGKLHTDKKQISVGFIGYPNVGKSSVINTLRSKKVCNVAPIAGETKVWQYITLMRRIFLIDCPGVVYPSE-D : 371
Dr gnl2    : SQEYPTLAFHASLTNSFGKGSLIQLLRQFGKLHSDKKQISVGFIGYPNVGKSSIINTLRSKKVCNVAPLAGETKVWQYITLMRRIFLIDCPGVVYPSD-D : 371
Dm ns2     : SAEYPTIAFHASLQHPFGKGALINLFRQLGKLHLDKKQISVGFIGYPNVGKSSVINALRSKKVCKVAPIAGETKVWQYITLMKRIFLIDCPGVVYPTA-E : 376
Sc NOG2    : SKERPTLAFHASITNSFGKGSLIQLLRQFSQLHTDRKQISVGFIGYPNTGKSSIINTLRKKKVCQVAPIPGETKVWQYITLMKRIFLIDCPGIVPPSSKD : 377
Ce ngp-1   : SKEMPTIAFHASINNSFGKGAVINLLRQFAKLHPDRPQISVGFIGYPNVGKSSLVNTLRKKKVCKTAPIAGETKVWQYVMLMRRIYLIDSPGVVYPQG-D : 386

                           *       420         *       440         *       460         *       480         *       500
Hs GNL2    : SETDIVLKGVVQVEKIKSPEDHIGAVLERAKPEYISKTYKIDSWENAEDFLEKLAFRTGKLLKGGEPDLQTVGKMVLNDWQRGRIPFFVKPPNAEPLVAP : 471
Mm Gnl2    : SETDIVLKGVVQVEKIKAPQDHIGAVLERAKPEYISKTYKIESWENAEDFLEKLALRTGKLLKGGEPDMLTVSKMVLNDWQRGRIPFFVKPPNAELPTDS : 471
Dr gnl2    : SETDIVLKGVVQVEKIRNPEDHIGAVLERAKAEYIQKTYRIPSWSSAEDFLEKLAFRTGKLLKGGEPDLPTVSKMVLNDWQRGRIPFFVKPPGVET--DE : 469
Dm ns2     : TDTEKVLKGVVRVELVTNPEDYVDSLLKRVRPEYISKNYKIEHWNTSTHFLEQLAQKTGKLLKGGEPDVTVTARMVLNDWQRGKLPFYVPPEGFAVPKSQ : 476
Sc NOG2    : SEEDILFRGVVRVEHVTHPEQYIPGVLKRCQVKHLERTYEISGWKDATEFIEILARKQGRLLKGGEPDESGVSKQILNDFNRGKIPWFVLPP-------- : 469
Ce ngp-1   : SETQIILKGVVRVENVKDPENHVQGVLDRCKPEHLRRQYGIPEFTDVDDFLTKIAIKQGRLLKGGDPDIVAVSKVVLNEFQRGKLPYFVPPPGCEER--- : 483

                           *       520         *       540         *       560         *       580         *       600
Hs GNL2    : QLLPSSSLEVVPEAAQNNPGEEVTETAGEGSESIIKEETEENSHCDANTEMQQILTRVRQNFGKINVVPQFSGDDLVPVEVSDLEEELESFSDEEEEEQE : 571
Mm Gnl2    : QLPPSSPLEVPTETTQNNPEEETTETEVERSDSITEKEPEGDCSQDRNSEMQQILARVRQNFGKINVGPQFSADDLVPVEMSDLED-LESSGEEEEQEQE : 570
Dr gnl2    : ENKAQAMLEMPEMESEMDELENRLEAQDVEEASSSQHE-EQKLLKKKKEEVQKLFNDVKQNFGKINVAPEFSEEDLVPVEMPDMMDASGSEDEENEEEEE : 568
Dm ns2     : EGKEE------EVVAEDPNEDAKSEAPTFVSES-----------VKKAREFKQI-----QDFRKIRVGLEYEQEDVKDLDHIDLELLEQQKAERAAKKKA : 554
Sc NOG2    : -------------------------------------------------------------------------------------EKEGEEKPKKKE : 481
Ce ngp-1   : ---------------------------------------------AKKDFSQAPINEMCADDDEQLPLDAQLELDDVARNGGPDEDDDEAIDDEEQEELTE : 539

                           *       620         *       640         *       660         *       680         *       700
Hs GNL2    : QQRDDAEES-SSEPEEE---------NVG-NDTKAVIKALDEKIAKYQKFLD-KAKAKKFSAVRISKGLSEKIFAKPEEQRKTLEEDVDDRAPSKKGKKR : 659
Mm Gnl2    : QPGEDAEEERSPDTQEE---------PVG-NDTKAVLRALDEKIAKYQRFLN-KAKAKKFSAVRISKDLSEKVFAKYKEEKKTSAEDSD-AAPTKKARKW : 658
Dr gnl2    : EEDEEKAEEETGEQQEEESSVSDSTPKAGKKSTQEVNKEMDEKIAKLKHFLD-RAKSKHFSAIRIPKGLSERVFTETVAKQNAAKNQQQ--KAVKQGKRR : 665
Dm ns2     : RIHNLGEEDEES------------------SDGADEFYSEDEYNEDLQRVVHKKAKSKKPQQLAITSSGKFRVAKIQPGDSDGAGSSDDDGPSTSKAP-- : 634
Sc NOG2    : VEKTA------------------------------------------------------------------------------------------------ : 486
Ce ngp-1   : IGSTCSGLTDLS----------------GISDLAGDLSDADDVADDDEKPSSSDSKTKKSKTDVASVRTRGKRGGKKANKAKLSGKTIVQAASEKKDK-- : 621

                           *       720         *       740         *       760         *
Hs GNL2    : KAQREEEQEHSNKAPRALTSKERRRAVRQQRPKKVGVRYYETHNVKNRNRNKKKTNDSEGQKHKRKKFRQKQ : 731
Mm Gnl2    : DAQMEEEP--SNKTQRMLTCKERRRAARQQQSKKVGVRYYETHNVKNRNRNKKKTSDSEGQKHRRNKFRQKQ : 728
Dr gnl2    : RA-VEDEP----AQPAKLTSKEKRRIERAQKVKKVGVRYYETHNVKNKNKNRKIPSD--GKKANRAKR---- : 726
Dm ns2     : ----------------RLTAKQKRSLERSQKRKKIGSNFYETTNVKNRNRNKKKDA---------------- : 674
Sc NOG2    : ------------------------------------------------------------------------ :   -
Ce ngp-1   : ------------------------SVVEFAKPGATKTNMWRKKLEKRRKVKHQK------------------ : 651
```

	Mm Gnl2	Dr gnl2	Dm ns2	Sc NOG2	Ce ngp-1
Hs GNL2	86%	66%	45%	34%	39%
Mm Gnl2		66%	46%	34%	39%
Dr gnl2			44%	35%	37%
Dm ns2				34%	41%
Sc NOG2					36%

B

```
                     *        20         *        40         *        60         *        80
Hs SEC13 : MVSVINTVDTSHEDMIHDAQMDYYGTRLATCSSDRSVKIFDV-RNGGQILIADLRGHEGPVWQVAWAHPMYGNILASCSY :  79
Mm Sec13 : MVSVMNTVDTSHEDMIHDAQMDYYGTRLATCSSDRSVKIFDV-RNGGQILIADLRGHEGPVWQVAWAHPMYGNILASCSY :  79
Dr sec13 : MVSVINTVDTSHEDMIHDAQMDYYGTRLATCSSDRSVKIFDV-KNGGQILVADLRGHEGPVWQVAWAHPMYGNILASCSY :  79
Dm sec13 : MVSLLQEIDTEHEDMVHHAALDFYGLLLATCSSDGSVRIFHS-RKN-NKALAELKGHQGPVWQVAWAHPKFGNILASCSY :  78
Sc SEC13 : MVVIAN----AHNELIHDAVLDYYGKRLATCSSDKTIKIFEV-EGETHKLIDTLTGHEGPVWRVDWAHPKFGTILASCSY :  75
Ce npp-20: MTTVRQRIDTQHRDAIHDAQLNIYGSRLATCGSDRLVKIFEVRPNGQSYPMAELVGHSGPVWKVSWAHPKYGGLLASASY :  80

                     *       100         *       120         *       140         *       160
Hs SEC13 : DRKVIIWREEN-GTWEKSHEHAGHDSSVNSVCWAPHDYGLILACGSSDGAISLLTYTGE-GQWEVKKINNAHTIGCNAVS : 157
Mm Sec13 : DRKVIIWKEEN-GTWEKTHEHSGHDSSVNSVCWAPHDYGLILACGSSDGAISLLTYTGE-GQWEVKKINNAHTIGCNAVS : 157
Dr sec13 : DRKVIIWKEEN-STWDKMYEYTGHDSSVNSVCWGPYDFGLILACGSSDGAISVLTCSGD-GHWDIKKINNAHTIGCNAVS : 157
Dm sec13 : DRKVIVWKSTTPRDWTKLYEYSNHDSSVNSVDFAPSEYGLVLACASSDGSVSVLTCNTEYGVWDAKKIPNAHTIGVNAIS : 158
Sc SEC13 : DGKVLIWKEEN-GRWSQIAVHAVHSASVNSVQWAPHEYGPLLLVASSDGKVSVVEFKEN--GTTSPIIIDAHAIGVNSAS : 152
Ce npp-20: DKKVIIWNEQQ-GRWQKAYEWAAHEASTTCVAFAPHQYGLMLASASADGDIGILRYDNSSNEWISSKIQKCHEQGVNSVC : 159

                     *       180         *       200         *       220         *       240
Hs SEC13 : WAPAVVPGSLIDHPSGQKPNYIKRFASGGCDNLIKLWKEEEDGQ-WKEEQKLEAHSDWVRDVAWAPSIGLPTSTIASCSQ : 236
Mm Sec13 : WAPAVVPGSLIDQPSGQKPNYIKKFASGGCDNLIKLWREEEDGQ-WKEEQKLEAHSDWVRDVAWAPSIGLPTSTIASCSQ : 236
Dr sec13 : WAPAVVPGSLIEQPTGQKPNYIKRFVSGGCDNLVKLWKEE-DGQ-WKEDQKLEAHSDWVRDVGWAPSIGLPTSTIASCSQ : 235
Dm sec13 : WCPAQAPDPAFDQRVTSRSAAVKRLVSGGCDNLVKIWRED-NDR-WVEEHRLEAHSDWVRDVAWAPSIGLPRSQIATASQ : 236
Sc SEC13 : WAPATIE----EDGEHNGTKESRKFVTGGADNLVKIWKYNSDAQTYVLESTLEGHSDWVRDVAWSPTV-LLRSYLASVSQ : 227
Ce npp-20: WAPGSAD-----------PAAKKRLVSAGNDKNVKIWAFDDATNEWILEKTLAGHTDFVREAAWCPVTNNGQHTIVSCGM : 228

                     *       260         *       280         *       300         *       320
Hs SEC13 : DGRVFIWTCDDASSNTWSPKLLH--KFNDVVWHVSWSITANILAVSGGDNKVTLWKESVDGQWVCISDVN------KGQG : 308
Mm Sec13 : DGRVFIWTCDDASGNMWSPKLLH--KFNDVVWHVSWSITANILAVSGGDNKVTLWKESVDGQWVCISDVN------KGQG : 308
Dr sec13 : DGRVFIWTCDDPAGNTWTAKLLH--KFNDVVWHVSWSITGNILAVSGGDNKVTLWKESVDGQWACISDVN------KGQG : 307
Dm sec13 : DRHVIVWSSN-ADLSEWTSTVLH--TFDDAVWSISWSTTGNILAVTGGDNNVTLWKENTEGQWIRINYESGTAIQSKQPS : 313
Sc SEC13 : DRTCIIWTQD-NEQGPWKKTLLKEEKFPDVLWRASWSLSGNVLALSGGDNKVTLWKENLEGKWEPAGEVH---------- : 296
Ce npp-20: EGNLVLFRTSNIETEEWKAKLLE--TAPCALYHSSFSPCGSFLSVAGDDNVITIWRENLQGQWIKVPRDN--------KE : 298

                     *       340         *       360
Hs SEC13 : SVSASVTEGQQNEQ----------------------------- : 322
Mm Sec13 : SVSASITEGQQNEQ----------------------------- : 322
Dr sec13 : AVS-SITDSQQSEQ----------------------------- : 320
Dm sec13 : HLPHSHSQQQQALQQHQQQAPSHPGPSSDSEHSSNLSNSQLSN : 356
Sc SEC13 : ---------Q--------------------------------- : 297
Ce npp-20: REGMSQAVGAPGAQR---------------------------- : 313
```

	Mm Sec13	Dr sec13	Dm sec13	Sc SEC13	Ce npp-20
Hs SEC13	96%	87%	50%	47%	40%
Mm Sec13		87%	51%	47%	39%
Dr sec13			51%	46%	39%
Dm sec13				39%	34%
Sc SEC13					35%

C

```
                   *        20         *        40         *        60         *        80
Hs RAB27A: MSDGDYD--------YLIKFLALGDSGVGKTSVLYQYTDGKFNSKFITTVGIDFREKRVVYRASGPDGATGRGQRIHLQL :  72
Mm Rab27a: MSDGDYD--------YLIKFLALGDSGVGKTSVLYQYTDGKFNSKFITTVGIDFREKRVVYRANGPDGAVGRGQRIHLQL :  72
Dr rab27a: MSDGDYD--------YLIKFLALGDSGVGKTSFLYQYTDSKFNSKFITTVGIDFREKRVVYKSSGPDGTAGRGQRIHIQL :  72
Hs RAB27B: MTDGDYD--------YLIKLLALGDSGVGKTTFLYRYTDNKFNPKFITTVGIDFREKRVVYNAQGPNGSSGKAFKVHLQL :  72
Mm Rab27b: MTDGDYD--------YLIKLLALGDSGVGKTTFLYRYTDNKFNPKFITTVGIDFREKRVVYDTQGADGASGKAFKVHLQL :  72
Dr rab27b: MTDGDYD--------YLIKLLALGDSGVGKTTFLYRYTDNKFNPKFITTVGIDFREKRVVYTTNSPNGTTGKTFKVHLQL :  72
Ce aex-6 : --MGDYD--------YLIKFLALGDSGVGKTSFLHRYTDNTFTGQFISTVGIDFKEKKVVYKSSR-GGFGGRGQRVLLQL :  69
Dm Rab27 : MRAAPPEPEPLQLAGSGEQFLVLGDSGVGKTCLLYQYTDGRFHTQFISTVGIDFREKRLLYNSRG------RRHRIHLQI :  74

                   *       100         *       120         *       140         *       160
Hs RAB27A: WDTAGQERFRSLTTAFFRDAMGFLLLFDLTNEQSFLNVRNWISQLQMHAYCENPDIVLCGNKSDLEDQRVVKEEEAIALA : 152
Mm Rab27a: WDTAGQERFRSLTTAFFRDAMGFLLLFDLTNEQSFLNVRNWISQLQMHAYCENPDIVLCGNKSDLEDQRAVKEEEARELA : 152
Dr rab27a: WDTAGQERFRSLTTAFFRDAMGFLLLFDLTNEQSFLNVRNWMSQLQTHAYCENPDIVLCGNKSDLEEQRAVKAENARELA : 152
Hs RAB27B: WDTAGQERFRSLTTAFFRDAMGFLLMFDLTSQQSFLNVRNWMSQLQANAYCENPDIVLIGNKADLPDQREVNERQARELA : 152
Mm Rab27b: WDTAGQERFRSLTTAFFRDAMGFLLMFDLTSQQSFLNVRNWMSQLQANAYCENPDIVLIGNKADLPDQREVNERQARELA : 152
Dr rab27b: WDTAGQERFRSLTTAFFRDAMGFLLMFDLTSQQSFLNVRNWMSQLQANAYCENPDIVLVGNKADLADQREVQEKQAKELA : 152
Ce aex-6 : WDTAGQERFRSLTTAFFRDAMGFILIFDITNEQSFLNIRDWLSQLKVHAYCEQPDIIICGNKADLENRRQVSTARAKQLA : 149
Dm Rab27 : WDTAGQERFRSLTTAFYRDAMGFLLIFDLTSEKSFLETANWLSQLRTHAYSEDPDVVLCGNKCDLLQLRVVSRDQVAALC : 154

                   *       180         *       200         *       220         *       240
Hs RAB27A: EKYGIPYFETSAANGTNISQAIEMLLDLIMKRMERCVD---------KSWIPEGVVRSNGHASTDQLS-EEKEKGACGC- : 221
Mm Rab27a: EKYGIPYFETSAANGTNISHAIEMLLDLIMKRMERCVD---------KSWIPEGVVRSNGHTSADQLS-EEKEKGLCGC- : 221
Dr rab27a: EKYGVPYFETSAANGENVSRAVEVLLDLIMKRMERCVD---------KSWIPDGTVRSNGHSTADLTEPHDPEQSKCAC- : 222
Hs RAB27B: DKYGIPYFETSAATGQNVEKAVETLLDLIMKRMEQCVE---------KTQIPDTVN----GGNSGNLDGEKPPEKKCIC- : 218
Mm Rab27b: EKYGIPYFETSAATGQNVEKSVETLLDLIMKRMEKCVE---------KTQVPDTVN----GGNSGKLDGEKPAEKKCAC- : 218
Dr rab27b: DKYGIPYFETSAATGSEVDKAVVTLLDLVMKRMEQCVE---------KPSADANTS----DGSS-KLSAAPAQQKKCAC- : 217
Ce aex-6 : DQLGLPYFETSACTSTNVEKSVDCLLDLVMQRIQQSVE---------TSSLPLSECR----GVSLDGDPSAASSYCANC- : 215
Dm Rab27 : RRYRLPYIETSACTGANVKEAVELLVGRVMERIENAACNREFSLLLTQSRCLPNIAYGQPEDLVRLHDRREEPCSRRNCR : 234


Hs RAB27A: -- :   -
Mm Rab27a: -- :   -
Dr rab27a: -- :   -
Hs RAB27B: -- :   -
Mm Rab27b: -- :   -
Dr rab27b: -- :   -
Ce aex-6 : -- :   -
Dm Rab27 : NC : 236
```

	Mm Rab27a	Dr rab27a	Hs RAB27B	Mm Rab27b	Dr rab27b	Ce aex-6	Dm Rab27
Hs RAB27A	95%	83%	70%	69%	67%	58%	47%
Mm Rab27a		84%	71%	70%	68%	57%	46%
Dr rab27a			70%	70%	68%	59%	47%
Hs RAB27B				94%	83%	59%	45%
Mm Rab27b					82%	58%	43%
Dr rab27b						59%	43%
Ce aex-6							44%

D

```
                          *        20         *        40         *        60         *        80
Hs PSMB10   : ---------MLKPALE-PRGGFSFENCQRNASLERVLP--GLKVPHARKTGTTIAGLVFQDGVILGADTRATNDSVVADKS :  69
Mm Psmb10   : ---------MLKQAVE-PTGGFSFENCQRNASLEHVLP--GLRVPHARKTGTTIAGLVFRDGVILGADTRATNDSVVADKS :  69
Dr psmb10   : ---------MLNTSTKTLTGGFSFENTRRNAVLEANLSEKGYSAPNARKTGTTIAGLVFKDGVILGADTRATDDMVVADKN :  72
Dr psmb10.2 : ------MALTSHVLEPSLCGFNFENATRNIVLENGAEEGKIKPPKALKTGTTIAGVVFKDGVVLGADTRATSDEVVADKM :  74
Hs PSMB7    : -------MAAVSVYAPPVGGFSFDNCRRNAVLEADFAKRGYKLPKVRKTGTTIAGVVYKDGIVLGADTRATEGMVVADKN :  73
Mm Psmb7    : -------MAAVSVFQPPVGGFSFDNCRRNAVLEADFAKKGFKLPKARKTGTTIAGVVYKDGIVLGADTRATEGMVVADKN :  73
Dr psmb7    : -------MATVSVCQYQPGGFSFENCRRNALLEADITKLGFSSPAARKTGTTICGIVYKDGVVLGADTRATEGMIVADKN :  73
Sc PUP1     : -----------------MAGLSFDNYQRNNFLAENSH----TQPKATSTGTTIVGVKFNNGVVIAADTRSTQGPIVADKN :  59
Ce pbs-2    : MATADVKSTVPHMDMDRGGAFDFSNCIRNQAMCKMGG----KAPKLTSTGTTIVAVAFKGGLVMGADSRATAGNIIADKH :  76

                          *       100         *       120         *       140         *       160
Hs PSMB10   : CEKIHFIAPKIYCCGAGVAADAEMTTRMVASKMELHALSTGREPRVATVTRILRQTLFRYQGHVGASLIVGGVDLTGPQL : 149
Mm Psmb10   : CEKIHFIAPKIYCCGAGVAADTEMTTRMAASKMELHALSTGREPRVATVTRILRQTLFRYQGHVGASLVVGGVDLNGPQL : 149
Dr psmb10   : CMKIHYIAPNIYCCGAGVAADAEVTTQMMSSNVELHSLSTGRPPLVAMVTRQLKQMLFRYQGHIGSSLIVGGVDVNGAQL : 152
Dr psmb10.2 : CAKIHYIAPNIYCCGAGTAADTEKTTDMLSSNLTIFSMNSGRNPRVVMAVNIIQDMLFRYHGMIGANLILGGVDCTGSHL : 154
Hs PSMB7    : CSKIHFISPNIYCCGAGTAADTDMTTQLISSNLELHSLSTGRLPRVVTANRMLKQMLFRYQGYIGAALVLGGVDVTGPHL : 153
Mm Psmb7    : CSKIHFISPNIYCCGAGTAADTDMTTQLISSNLELHSLTTGRLPRVVTANRMLKQMLFRYQGYIGAALVLGGVDVTGPHL : 153
Dr psmb7    : CSKIHYISPNIYCCGAGTAADTEMTTQIISSNLELHSLSTGRLPRVATANRMLKQMLFRYQGYIGAALVLGGVDCTGPHL : 153
Sc PUP1     : CAKLHRISPKIWCAGAGTAADTEAVTQLIGSNIELHSLYTSREPRVVSALQMLKQHLFKYQGHIGAYLIVAGVDPTGSHL : 139
Ce pbs-2    : CEKVHKLTESIYACGAGTAADLDQVTKMLSGNLRLLELNTGRKARVITALRQAKQHLFNYQGYIGAYLLIGGVDPTGPHL : 156

                          *       180         *       200         *       220         *       240
Hs PSMB10   : YGVHPHGSYSRLPFTALGSGQDAALAVLEDRFQPNMTLEAAQGLLVEAVTAGILGDLGSGGNVDACVITK-TGAKLLRTL : 228
Mm Psmb10   : YEVHPHGSYSRLPFTALGSGQGAAVALLEDRFQPNMTLEAAQELLVEAITAGILSDLGSGGNVDACVITA-GGAKLQRAL : 228
Dr psmb10   : YSVYPHGSYDKLPFLTMGSGAASAISVFEDRYKPNMELEEAKQLVRDAITAGIFCDLGSGSNVDLCVITD-KKVDYLRTY : 231
Dr psmb10.2 : YTVGPYGSMDKVPYLAMGSGDLAAMGILEDRFKVNMDLEQAKALVSDAIQAGIMCDLGSGNNIDLCVITK-EGVDYIRPH : 233
Hs PSMB7    : YSIYPHGSTDKLPYVTMGSGSLAAMAVFEDKFRPDMEEEEAKNLVSEAIAAGIFNDLGSGSNIDLCVISK-NKLDFLRPY : 232
Mm Psmb7    : YSIYPHGSTDKLPYVTMGSGSLAAMAVFEDKFRPDMEEEEAKKLVSEAIAAGIFNDLGSGSNIDLCVISK-SKLDFLRPF : 232
Dr psmb7    : YSIYPHGSTDKLPYVTMGSGSLAAMAVFEDRYRPDMEEEDAKSLVRDAIAAGIFNDLGSGSNIDVCVITK-GKVDYLRPH : 232
Sc PUP1     : FSIHAHGSTDVGYYLSLGSGSLAAMAVLESHWKQDLTKEEAIKLASDAIQAGIWNDLGSGSNVDVCVMEIGKDAEYLRNY : 219
Ce pbs-2    : YMCSANGTTMAFPFTAQGSGSYAAITILERDFKVDMTKDEAEKLVQRALEAGMHGDNASGNSLNLVIIEP-SETVFKGPI : 235

                          *       260         *       280         *
Hs PSMB10   : SSPTEPVKR---------SGRYHFVPGTTAVLTQTVKPLTLELVEETVQAMEVE--- : 273
Mm Psmb10   : STPTEPVQR---------AGRYRFAPGTTPVLTREVRPLTLELLEETVQAMEVE--- : 273
Dr psmb10   : DQPVHKNQR---------GGTYRYKPGTTAVLSKTVTPLTLDVVDESVHVMDTE--- : 276
Dr psmb10.2 : KESPYNYKR---------QAKYKYKSGTTPILTKTVNKLELDLVQETVQMMETSASS : 281
Hs PSMB7    : TVPNKKGTR---------LGRYRCEKGTTAVLTEKITPLEIEVLEETVQTMDTS--- : 277
Mm Psmb7    : SVPNKKGTR---------LGRYRCEKGTTAVLTEKVTPLEIEVLEETVQTMDTS--- : 277
Dr psmb7    : DIANKKGVREDKLDGRPVTGSYRYKHGTTGVLSKAVTPLNLDMVEESVQTMDTS--- : 286
Sc PUP1     : LTPNVREEK---------QKSYKFPRGTTAVLKESIVN-ICDIQEEQVDITA----- : 261
Ce pbs-2    : VPEFCKRPE-------PNDLVYKFQAGATKVLKHKTYK------YDVVESMDITH-- : 277
```

	Mm Psmb10	Dr psmb10	Dr psmb10.2	Hs PSMB7	Mm Psmb7	Dr psmb7	Sc PUP1	Ce pbs-2
Hs PSMB10	88%	63%	51%	55%	56%	53%	44%	35%
Mm Psmb10		61%	50%	55%	56%	53%	43%	34%
Dr psmb10			55%	62%	62%	65%	45%	34%
Dr psmb10.2				55%	56%	55%	42%	36%
Hs PSMB7					96%	76%	51%	40%
Mm Psmb7						77%	51%	41%
Dr psmb7							48%	37%
Sc PUP1								37%

E

```
                      *        20         *        40         *        60         *        80         *       100
Ce noah-2: ---------MWGVIFLLLSIVPAAQSVFECSSHETTAFVRIPRARLDG--TPVVISTAG---HDLTCAQYCRNNIEPTTGAQRVCASFNFD--------- :   77
Dm nompA : MRPRKGIHVLLTTLVVSLSLSKINGQTTCKNGLGRVLYERLPNQQLQGYDDDVVRDTAPPFRVLEKCQDLCLRDRSGSNNLVRTCTSFDFQPGSRITSFG :  100

                      *       120         *       140         *       160         *       180         *       200
Ce noah-2: -----GRETCYFFDDAATPAGTSQLTANPSANNFYYEKTCIPNVSAHEACTYRSFSFERARNTQLEG--FVKKSVTVENREHCLSACLKEKEFVCKSVNF :  170
Dm nompA : GNSEYEESLCYLTSEQAGPEGIGSLMLVP--NSVHFNEICLTSSRPERECPSRRYVFERHPRKKLKLPISDIKEITAANRSDCEDKCLNEFSFVCRSANF :  198

                      *       220         *       240         *       260         *       280         *       300
Ce noah-2: HYDTSLCELSVEDKRSKPTHVRMSEKIDYYDNNCLSRQNRCGPSGGNLVFVKTTNFEIR-YYDHTQSVEAQESYCLQKCLDSLNTFCRSVEFNPKEKNCI :  269
Dm nompA : DSTMRSCTLSRFTRRTHPELMEDDPNSDYLENTCLNAERRCD---GLAVFVKEENKRLGGPFEVDIFNNMTLEECQTMCLRAEKYFCRSVEFDDQSKQCI :  295

                      *       320         *       340         *       360         *       380         *       400
Ce noah-2: VSDEDTFSRADQQGQVVG--KDYYEPICVAADLS-------------SSTCRQQAAFERFIGSSIEGEVVASAQGVTISDCISLCFQN--LNCKSINYDR :  352
Dm nompA : LSEEDSISQKDDISISSSPTHHFYDLVCLDNQRANDYPDNSVTSHLFSSGRRPDTAFQRYRNSRLGGEFHSEITGRSLSECLDECLRQTSFQCRSAVYSD :  395

                      *       420         *       440         *       460         *       480         *       500
Ce noah-2: TASSCFIYAVGRQD------------------------------------------------------------------------------------- :  366
Dm nompA : RFRTCRLSRYNQKDGMRIIYDADYDYYENLMLNVVGGGADGDGGGHGGSSDGKRPGDQSGSNWRQPNKHDDRYGSGGSSVGGGSHGGTGSGGSRLPPGEG :  495

                      *       520         *       540         *       560         *       580         *       600
Ce noah-2: ---------------------------------------------------------------------------ANIKAN--------------------------- :  372
Dm nompA : VDYGRPYDRYPDIAGNEYDRNPYGGDRDRDRYPPDRYGSRYPTGGDGIGYNRPYDRFPDDYDRYPAGAGVNGDRDRERDRDRYPVVGDRDRYPGAVDRDR :  595

                      *       620         *       640         *       660         *       680         *       700
Ce noah-2: ---------------------------------------------------------------------------------------------------- :    -
Dm nompA : YPGVRDRDRYPEPYPPERYADRYGDRRYPERERDRDRDRLPYRPLPYPGINDNSLPSDLPHTRPYPTDDDAPFRPYGYGGGRYGENRYEGRYPPRFPPSR :  695

                      *       720         *       740         *       760         *       780         *       800
Ce noah-2: ---------------------------------------------------------------------------------------------------- :    -
Dm nompA : ERDPVGGYTGRDAPDSIFPDRRYRPSSMDSPRYPYLPDSRGPPGRYDDIVHSARRPEPDSAKRYPPAPIAPTGSSSKYASTPNRFPVGNDRYPIDIYKYG :  795

                      *       820         *       840         *       860         *       880         *       900
Ce noah-2: ----------------PSMDYYEFNCESQFGG-------------------------------------------------------------------- :  388
Dm nompA : NRPGPNDLGRRPGLERPPPPFYDYDYEERYGDRYGPYDREYDGPPGRRPPSGGPYGRYDSPFNRPYGGNGLDDRPLPLPGLGLSHPPTPYGGGGAGHVGV :  895

                      *       920         *       940         *       960         *       980         *      1000
Ce noah-2: ---------------------------------------------------------------------------------------------------- :    -
Dm nompA : GVGVNSGPPRPPITRCEESDNFKQIAARHKMRRHFVRRALIVPSLIQCERECIESRDFVCRSFNYRDSAASGYEDRDRDRDRDSPNCELSDRDSRELDIH :  995

                      *      1020         *      1040         *      1060         *      1080         *      1100
Ce noah-2: ---------------------------MALCTNEGIRFIVNTKEPYTGAIYAAERFSTCSQVVENAKQISITFPPPTVSSDCGTVIRDGKMEALVVVSLD :  461
Dm nompA : DPGTFDASNYDFYERSIGRSDGECMDVTQTCNEEGMEFTIRTPEGFLGRIYTYGFYDRCFFRGNGGTVNVLRLSGPQGYPDCGTQRYGDTLTNIVVVQFS : 1095

                      *      1120         *      1140         *      1160         *      1180         *      1200
Ce noah-2: GVLPHQVTTEWDRFYRVSCDVSMDKMVKEGSVVVTTIYEASSQNTTVLDVATPPPVSAELQILNQLEEPLHKASIGDPILLVITSEQAGPHNMMVTECTA :  561
Dm nompA : ----DNVQTSRDKRYNLTCIFRGPGEAVVSSGYIG-AGSGSPIPIEYLPAENTLSSKVRLSIIYQ-GRPTTTIAVGDPITFRLEAQDGYNHVTDIFATNV : 1189

                      *      1220         *      1240         *      1260         *      1280         *      1300
Ce noah-2: TRVGGFGDTVPFTLIENGCPRYPALVGPVEQDFDKNRLKSDLRAFRLDGS---------YDVQIVCSIMFCAGPNG------------------------ :  628
Dm nompA : VARDPYSGRSIQLIDRFGCPVDPFVFPELDKLRDGDTLEARFNAFKIPESNFLVFEATVRSCREGCQPAYCPGPAGRQEPSFGRRRRSLNTTEIPEPEAL : 1289

                      *      1320         *      1340         *      1360         *      1380         *      1400
Ce noah-2: ---------------CPVSNCLDSGTNELFMSHGRKKRSADLEAGETEEKLSAIIRVFAKGEDEEEMEMANNTMMTSMSDSTELLCIAEPFFVSSVVSLS :  713
Dm nompA : ALEGSSQLEASTLDEVTVVNSTTVSATLGQVPLNETQLGEKTKETEEPEQVREMIEVFETREEIEKESYPR--KLVAPVETVCMTPAEYHGLITAIILLM : 1387

                      *      1420         *      1440         *      1460         *      1480         *      1500
Ce noah-2: VLCFALSAIIAIWGCHSLHSKPVKQVAA------------------------------------------------------------------------ :  741
Dm nompA : ILLFSITLVAGLGYRRYWKSISKNRLVDRHSPIHSLGHSHSSIRTHERFTEIGHMPNLNGGGGGAAAGTGGGANQSASNRASNAFRTNMSMFGGSLHKTF : 1487

                      *      1520         *      1540         *      1560         *
Ce noah-2: ---------------------------------------------------------------------- :    -
Dm nompA : ATGNLARMCQLPVINPMRSTNQSSHQFEDPSEPIYTDPSLFERSRQVADHSVTHPQNEFPTTVRNYRCEV : 1557
```

Percentage Identity: 10%
