# Supplementary material for: Identification and characterization of novel factors that act in the nonsense-mediated mRNA decay pathway in nematodes, flies and mammals
Source: EMBO Rep. 2014 Dec 1;16(1):71–8. doi: 10.15252/embr.201439183 (PMC4304730; doi:10.15252/embr.201439183)
Supplement: Supplementary file 5 [file embr0016-0071-sd5.pdf]

Identification and characterization of novel factors that act in the nonsense-mediated mRNA decay pathway in nematodes, flies and mammals

Angela Casadio[1], Dasa Longman[1*], Nele Hug[1], Laurent Delavaine[1], Raúl Vallejos Baier[2], Claudio R. Alonso[2] and Javier F. Cáceres[1*]

Supplementary Information

Supplementary Methods

Cell culture and siRNA-mediated depletion

HeLa cells were grown in Dulbecco's modified Eagle's medium (Life Technologies) supplemented with 10% fetal calf serum and incubated at 37℃ in the presence of 5% CO_2. For siRNA-mediated depletions, HeLa cells were transfected using a pool of four siRNAs (OnTarget Plus siRNA, Thermo Scientific) against each gene tested and were transfected using DharmaFECT 1 (Thermo Scientific) following manufacturer's instructions. Depletion of *UPF1* was achieved by using a combination of two individual siRNAs previously described in [1,2]. Catalogues numbers of OnTarget Plus siRNA pools: *GNL2* (L-020392-010005), *SEC13* (L-012351-00-0005), *RAB27A* (L-004667-00-0005), *RAB27B* (L004228-00-0005), *PSMB7* (L-006021-00-0005), *PSMB10* (L-006019-00-0005), *UPF2* (L-012993-01-0005), scramble (D-001810-02-20). *UPF1* siRNAs sequence: hUPF1-I GAGAATCGCCTACTTCACT and hUPF1-II GATGCAGTTCCGCTCCATT. Transfections were performed in six well plates in four biological replicates; cells were seeded at 15% confluency at day 0 and transfected on day 1 with 5 µM siRNA. On day 3, cells were passaged in order to reach the right confluency level for a second round of transfection on day 4. Cells were then collected 2 days after the second round of transfection. Cells treated with siRNAs against *PSMB7* and *PSMB10* were collected 1 day after the second round of transfection. Total RNA was isolated using the Qiagen RNeasy kit following manufacturer's instruction. The quality of RNA was assessed by Agilent 2100 Bioanalyzer using

RNA 6000 Nano Kit, and NanoDrop 8000 spectrophotometer. The efficiency of the knockdown was assessed by RT-qPCR.

Half-life mRNA analysis

HeLa cells stably expressing *HBB* gene, wild type or NS39, were mock-depleted or depleted of *GNL2* or *SEC13* for 5 days, as described above. On day 5, Actinomycin D (2 µg/ml) was added to the depleted cells. Total RNA was isolated from cells treated with Actinomycin D for 0.5, 1, 2, 4, 6, and 8 hours. The quality of RNA was assessed by Agilent 2100 Bioanalyzer using RNA 6000 Nano Kit, and NanoDrop 8000 spectrophotometer. For each time point, the mRNA levels of the *HBB* reporters were monitored by qRT-PCR and normalized to *POLR2J* and *ACTB* reference genes. The values shown are the average fold-change (mean ± SEM) from three independent experiments relative to the first time point. The decay curve was calculated using GraphPad Prism software (exponential one phase decay curve) and displayed on log2 y-axis. For the half-life analysis of ARHGEF18 mRNA, untransfected Hela cells were treated as above.

cDNA synthesis and RT-PCR (*C. elegans*)

cDNA was synthesised starting from 500 ng of RNA using Transcriptor Universal cDNA master (Roche) according to manufacturer's instructions. Quantitative RT-PCR was performed using the SYBR Green system (Roche) following manufacturer's instructions. The expression of GFP was normalized to that of the reference gene *ama-1*. The sequence of the primers used is listed in Supplementary Table S1.

cDNA synthesis and quantitative RT-PCR (human cells)

cDNA was synthesized from 1 µg of total RNA using Transcriptor Universal cDNA master (Roche) according to manufacturer's instructions. Quantitative RT-PCR was performed using the probe system (Roche) following

manufacturer's instructions. The expression of β-globin was normalized to the geometric mean of two reference genes (*ACTB* and *POLR2J*). To check depletion levels of the putative factors and of the positive control *UPF2*, their mRNA level was normalized to the geometric mean of the two reference genes. The expression of *ARHGEF18*, *BMP2* and *TMC7* was normalized to the geometric mean of two reference genes (*ACTB* and *POLR2J*). To analyze the half-life of the two β-globin reporters and of *ARHGEF18* mRNAs, the expression of their RNAs was measured at the indicated time points and normalized to the geometric mean of two reference genes (*ACTB* and *POLR2J*). To analyze the feedback loop, mRNAs corresponding to known NMD factors were normalized to the geometric mean of two reference genes (*ACTB* and *POLR2J*). The sequences of the primers used for RT-qPCR and catalogue numbers for the Roche assays are listed in the Supplementary Table S1.

Translation inhibition assay

HeLa cells were depleted with siRNA for 5 days, as described previously. After siRNA treatment overall translation activity was measured as described by [3]. Cells were labelled for 1h with [^{35}S]-Met/Cys. Afterwards, labeled protein extract concentration was determined using Bradford assay. Equal amount of protein was separated on 4-12% Bis-Tris gels (Life Technologies). After gel drying, the gels were stained using Coomassie Colloidal Blue (Life Technologies). Radioactivity in each sample was determined using autoradiography, radioactive signals and Coomassie staining signals were quantified with ImageQuantTL software (GE Healthcare).

Immunoprecipitation and Western Blotting

HEK293T cells were transiently transfected with Flag-tagged UPF1 or Flag empty vector (F-EV) together with either T7-tagged GNL2 or T7 empty vector (T7-EV). GNL2 cDNA was cloned by PCR amplification (Forward Primer: CTGCTAGCATGGTGAAGCCCAAGTACAA and Reverse Primer: CGACTGTGATCATTACTGCTTTTGTCTGAATTTTTTGCG) and ligated into pCG-T7 vector. Flag-UPF1 was previously described [4]. In total, 10ng of each plasmid together with 2 µg of carrier DNA per well was transfected in 6-well plates, expanded into 10 cm dishes and harvested 48hrs post transfection. All subsequent steps were carried out at 4ºC. Cells were washed 2x in PBS and 10^7 cells were lysed in 1 ml of IP buffer (10mM Tris HCl pH8, 150mM NaCl, 1% NP-40, 0.2% Sodium Deoxycholate, 1mM EDTA, 1mM DTT, protease inhibitors (Roche)) for 20 min.

For RNase treatment half of protein extract was incubated with 20 μg/ml RNase A (ThermoScientific) for 20 min. Cell extracts were spun 20 min at full speed and supernatants were incubated with Anti-Flag M2 magnetic beads (Sigma) o/n at 4ºC. Beads were washed 5x in IP buffer and resuspended in LDS loading buffer supplemented with reducing agent (Life Technologies). Samples were incubated at 94ºC for 5 min, and subjected to SDS/polyacrylamide gel electrophoresis followed by Western blotting using rabbit anti-GNL2 antibody (1:500) (Sigma), rabbit anti-PABP1 antibody (1: 1000) (Cell Signaling), or goat anti-RENT1 (UPF1) antibody (1:3000) (Bethyl). Proteins were visualized by Fluorchem (Protein Simple).

Immunostaining experiments in *Drosophila* embryos

Late stage 16 embryos were collected, dechorionated in bleach 50% and fixed in 1x PBS formaldehyde 4% according to standard protocols. Nuclei were stained with DAPI, neurons of the PNS were labelled with a mouse monoclonal anti-futsch antibody (22C10, Hybridoma bank) and GFP was detected in fixed tissue using a rabbit polyclonal anti-GFP antibody (Life Technologies). Secondary antibodies were Alexa Fluor 555 Donkey Anti-Mouse IgG (H+L) and Alexa Fluor 488 Donkey Anti-Rabbit IgG (H+L), respectively (both from Life Technologies).

Embryos were mounted in Vectashield reagent (Vector Labs) and unsaturated images of GFP staining of late stage 16 embryos were taken using a DM6000 microscope and LAS software (Leica Microsystems), using identical settings between experimental and control samples. Images were imported into ImageJ (NIH), converted into jpeg format, exported to Adobe Photoshop PS6 (Adobe Systems Incorporated) and average levels of pixel intensity extracted from the green channel were measured in abdominal PNS fields comparing identical areas (250 μm^2) in Upf1-RNAi, nompA-RNAi and wild-type (no RNAi) control embryos. After subtraction of background, ratios between GFP expression in control and genetically induced RNAi treatments were determined and plotted using GraphPad Prism (GraphPad Software).

Supplementary References

1. Paillusson A, Hirschi N, Vallan C, Azzalin CM, Mühlemann O (2005) A GFP-based reporter system to monitor nonsense-mediated mRNA decay. *Nucleic Acids Res* **33**: e54.
2. Azzalin CM, Lingner J (2006) The human RNA surveillance factor UPF1 is required for S phase progression and genome stability. *Curr Biol* **16**: 433–439.
3. Chazal P-E, Daguenet E, Wendling C, Ulryck N, Tomasetto C, Sargueil B, Le Hir H (2013) EJC core component MLN51 interacts with eIF3 and activates translation. *Proc Natl Acad Sci U S A* **110**: 5903–5908.

4. Hug N, Cáceres JF (2014) The RNA Helicase DHX34 Activates NMD by Promoting a Transition from the Surveillance to the Decay-Inducing Complex. *Cell Rep* **8**: 1845–1856.

Legends for Supplementary Figures

Figure S1. **Schematic representation of the novel NMD proteins.** (**A**) The *C. elegans* NGP-1 protein and its human homolog GNL2 are characterized by a conserved N-terminal NGP1NT domain (yellow) and by a GTPase domain (green). The five G-motifs (black) that are typical of this class of GTPases are indicated. (**B**) The *C. elegans* NPP-20 protein and its human homolog SEC13 are characterized by the presence of six WD40 domains (orange). (**C**) The *C. elegans* AEX-6 protein and its two human homologs RAB27A and RAB27B are small GTPase members of the RAB subfamily and are characterized by a highly conserved RAB domain (purple). (**D**) The *C. elegans* PBS-2 protein and its two human homologs PSMB7 and PSMB10 are characterized by the presence of a proteasome domain (light blue). The two human proteins also contain a proteasome beta subunits C-terminal domain (dark blue). (**E**) The *C. elegans* NOAH-2 protein and its *D. melanogaster* homolog nompA are characterized by 4 PAN_AP-domains (red) and by a ZP-domain (dark orange). Both proteins contain an N-terminal signal peptide sequence (yellow) and a C-terminal transmembrane (TM) signal sequence (black). Predicted conserved protein domains were generated using SMART (http://smart.embl-heidelberg.de). For each conserved protein % of identity is indicated.

Figure S2. **Alignment of orthologous proteins identified in the RNAi screen.** (a) *ngp-1* (b) *npp-20* (c) *aex-6* (d) *pbs-2* and (e) *noah-2*. Sequences were aligned using CLUSTAL 2.1, and output was produced using Genedoc (version 2.7.00). Residues highlighted in black: 100% homology, dark grey: 80% homology, pale grey: 60% homology. Similarity is defined by a scoring matrix (PAM-65), which reflects the degree to which residues are interchangeable or equivalent during evolution. The table below each alignment indicates % identity of proteins across species. Hs: human; Mm: mouse; Dr: zebrafish; Dm; fruitfly; Ce: *C.elegans*; Sc; yeast

Figure S3. **Role of the newly identified human homologs in NMD.**

(**A, B**) HeLa cells stably expressing wild-type β-globin reporter (**A**) or β-globin NS39 NMD-reporter (**B**) were mock-depleted, or depleted of the indicated factors for four days. The level of the β-globin mRNA was monitored by quantitative RT-PCR and normalized to two reference genes (*POLR2J* and *ACTB*). The values shown are the average fold-change (mean ± SEM) from four independent experiments relative to mock-depleted cells (control). Statistical analysis was performed using the Mann-Whitney test for non-parametric distributions. * $p < 0.05$. (**C**) The level of mRNA of the indicated factors following siRNA-mediated depletion in HeLa cells were measured by qRT-PCR. These samples were used in further analysis shown in Figures 4 and S3. All values are the average of at least three biological replicas showing +/- S.E.M. (**D**) *GNL2* and *SEC13* regulate the half-life of *ARHGEF18* mRNA. HeLa cells were mock-depleted or depleted of *GNL2* or *SEC13* for 5 days and treated with Actinomycin D as indicated. Samples were collected after the indicated times and the mRNA level of *ARHGEF18* was monitored by qRT-PCR and normalized to *POLR2J* and *ACTB* reference genes. The values shown are the average fold-change (mean ± SEM) from three independent experiments relative to the first time point. (**E)** Depletion of new NMD factors does not affect global translation. HeLa cells were depleted of *UPF2*, *GNL2* and *SEC13* and metabolic labeling was carried out by incubation with [^{35}S]-Met/Cys for 1h. Total protein extracts were analyzed by Coomassie staining. The incorporation of [^{35}S]-Met/Cys was determined by autoradiography. (**F**) Bar charts showing quantification of the [^{35}S]-Met/Cys incorporation normalized to the total protein concentration. Autoradiograph signals and Coomassie staining were analyzed using the ImageQuant TL software (GE Healthcare). All values represent the average fold-change (mean ± SD) from at least two independent experiments relative to mock-depleted cells. siRNA mediated depletion of NMD factors were measured as described before by RT-qPCR (depletion (mean ± SD) was 0.22±0.03 for *UPF2*; 0.12±0.01 for *GNL2* and 0.32±0.02 for *SEC13*).

Figure S4. GNL2 co-immunoprecipitates with the core NMD factor UPF1. Immunoprecipitation of Flag-tagged UPF1 co-purified T7-tagged GNL2 in an RNA-independent manner. As a control of RNase A treatment, UPF1 interaction with PABP1 protein was lost in the absence of RNA. All tagged proteins were expressed at near endogenous levels. F-EV: Flag-empty vector; T7-EV: T7-empty vector. The asterisk indicates an unspecific band.

Supplementary Table S1. List of primers and PCR assays used

Gene	Primers	Sequence	UPL Probe Number (Roche)	Assay ID (Roche)
Ce *ama-1*	Forward	TCTGTCACATCACCGTACAA	n.a.	n.a.
	Reverse	CAGAGAGTATCCTGGACGAT		
GFP	Forward	TGAAGGTGATGCAACATACG	n.a.	n.a.
	Reverse	CGGGCATGGCACTCTTGAAA		
Hs *ACTB*	Forward	AGAGCTACGAGCTGCCTGAC	9	n.a.
	Reverse	CGTGGATGCCACAGGACT		
Hs *POLR2J*	Forward	CTGTGAGCCCCGTTCCTAC	1	n.a.
	Reverse	GTCGGTGTCAGGGTGAGG		
Hs *HBB*	Forward	n.a.	n.a.	102141
	Reverse			
Hs *UPF2*	Forward	n.a.	n.a.	137425
	Reverse			
Hs *GNL2*	Forward	n.a	n.a.	144594
	Reverse			
Hs *SEC13*	Forward	n.a.	n.a.	138390
	Reverse			
Hs *RAB27A*	Forward	n.a.	n.a.	114763
	Reverse			
Hs *RAB27B*	Forward	n.a.	n.a.	115465
	Reverse			
Hs *PSMB7*	Forward	TGATAAGTTGCCTTATGTCACCA	36	n.a.
	Reverse	TCCTCCATGTCTGGCCTAAA		
Hs *PSMB10*	Forward	CATCGCCCCCAAAATCTAC	19	n.a.
	Reverse	CCATCCGTGTGGTCATCTC		
Hs *UPF1*	Forward	n.a.	n.a	115402
	Reverse			
Hs *SMG1*	Forward	n.a.	n.a	106402
	Reverse			
Hs *SMG5*	Forward	n.a	n.a.	128927
	Reverse			
Hs *TMC7*	Forward	n.a.	n.a	142251
	Reverse			
Hs *ARHGEF18*	Forward	ATCAGGGCGCTTGAAAGATA	18	n.a.
	Reverse	TTCTTGTAGCAGCAAAAGTACGTC		
Hs *BMP2*	Forward	GACTGCGGTCTCCTAAAGGTC	49	n.a.
	Reverse	GGAAGCAGCAACGCTAGAAG		
