# Supplementary material for: Identification and characterization of novel factors that act in the nonsense-mediated mRNA decay pathway in nematodes, flies and mammals
Source: EMBO Rep. 2014 Dec 1;16(1):71–8. doi: 10.15252/embr.201439183 (PMC4304730; doi:10.15252/embr.201439183)
Supplement: Supplementary file 6 [file embr0016-0071-sd6.pdf]

EMBO
reports

Manuscript EMBOR-2014-39183

Identification and characterization of novel factors that act in the nonsense-mediated mRNA decay pathway in nematodes, flies and mammals

Angela Casadio, Dasa Longman, Nele Hug, Laurent Delavaine, Raul Vallejos Baier, Claudio R. Alonso and Javier F. Caceres

Corresponding author: Javier F. Caceres and Dasa Longman, MRC, Institute of Genetics and Molecular Medicine, University of Edinburgh

Review timeline:

Submission date:	17 June 2014
Editorial Decision:	15 July 2014
Revision received:	13 October 2014
Editorial Decision:	23 October 2014
Revision received:	30 October 2014
Accepted:	03 November 2014

Transaction Report:

(Note: With the exception of the correction of typographical or spelling errors that could be a source of ambiguity, letters and reports are not edited. The original formatting of letters and referee reports may not be reflected in this compilation.)

Editor: Esther Schnapp

1st Editorial Decision 15 July 2014

Thank you for the submission of your research manuscript to EMBO reports. I am back in the office now and have taken over its handling, as it falls into the RNA subject category that I handle at our journal. We have now received the full set of referee reports that is copied below.

As you will see, the referees acknowledge that the identification of new NMD candidate proteins is potentially interesting. However, referee 2 points out that a role for the identified proteins in NMD must be strengthened (by investigating mRNA half-life and endogenous NMD substrates), and that obvious indirect effects need to be excluded. Referees 1 and 3 further mention that it should be investigated whether some of the candidate NMD proteins have overlapping functions, and that certain controls need to be added or better explained.

Given these constructive comments, we would like to invite you to revise your manuscript with the understanding that the referee concerns (as mentioned above and in their reports) must be fully addressed and their suggestions taken on board. Given that only a relatively small number of concerns was raised, I did not ask the referees to cross-comment on each others' reports, as I think

© European Molecular Biology Organization

that all concerns need to be addressed. Acceptance of the manuscript will depend on a positive outcome of a second round of review. It is EMBO reports policy to allow a single round of revision only and acceptance or rejection of the manuscript will therefore depend on the completeness of your responses included in the next, final version of the manuscript.

Revised manuscripts should be submitted within three months of a request for revision. Also, the revised manuscript may not exceed 30,000 characters (including spaces, references and figure legends) and 5 main plus 5 supplementary figures. The current manuscript text slightly exceeds our limits, and the text therefore needs to be somewhat shortened. Please note that the materials and methods section cannot be shortened further.

Please add a scale bar to the images in figure 3 and define its length in the figure legend.

I look forward to seeing a revised version of your manuscript when it is ready. Please let me know if you have questions or comments regarding the revision.

REFEREE REPORTS:

Referee #1:

The authors use the same screen but a different C. elegans RNAi library than previously used to identify new NMD factors. This library includes dsRNAs that downregulate 1,736 genes not downregulated in the previous library and targets ~ 55% of the genome. The authors found and confirmed the existence of five new NMD factors:
C. elegans NGP-1 = human GNL2, a putative GTPase - yeast Nop2p, involved in ribosome biogenesis, processing and possibly nuclear export of pre-60 ribosomal particles.
C. elegans NNP-20 = human SEC13 and is constituent of the ER and NPC.
C. elegans AEX-6 involved in defecation = human RAB27A and B, member of the Rab smll GTPases
C. elegans PBS-2 is a member of the proteosome B-type family = human PSMB7 and 10

C. elegans NOAH-2 is the only of the five not conserved and has been shown to be required for embryonic and larval development.
The authors show that worms defective in NGP-1 are largely arrested in L1-L2 stage; worms defective in in NPP-20 are largely arrested in L2-L3; defects in AEX-6 progress with only a mild NPP-20-like defect; worms defective in in PBS-2 are arrested in L2; and in NOAH-2 in L2-L3.

The Drosophila ortholog of NOAH-2, NOMPA, which is expressed in the embryonic peripheral nervous system, was shown to function in NMD. So were GLN2 and SEC13 in human cells using NMD reporters.

This submission certainly adds to the field. It is a bit of a mish-mash. Some explanation for why different species were used to assay for roles of the newly discovered C. elegans NMD factors is warranted. Additional comments are below.

Comments
Page 9. Why wasn't C. elegans used to study NOAH-2 function in NMD prior to larval arrest.

Figure 4 legend. "or depleted of UPF1....PSMB7 and PSMB10 using a pool of four siRNAs". What was done was probably not what is written since each protein was probably downregulated using a pool of siRNAs. Also, the use of pools of siRNAs raises a concern for off-target effects.

Figure 4 legend. What were HBB mRNAs from the wild-type and NS39 reporters normalized to so that they could be compared? Also, what were the levels of endogenous NMD targets normalized to?

Pages 10-11. "co-immunoprecipitated" would be a better term than "interacted".

© European Molecular Biology Organization

Figure 4. Using tagged proteins is fine but their level relative to endogenous protein should be approximately the same. Was this true for Flag-tagged UPF1, T7-tagged GLN2 or HA-tagged SEC13?

Figure 4 legend. Please define "EV" as empty vector so there is no confusion.

Figure 4C. What happened to the WB using anti-Flag? There is no band in the IP - possibly a lighter exposure is required.

Figure 4E. The key should include "siRNA" since that is what were used. And, the x axis should include "mRNA".

Page 11. Last paragraph. How to the authors explain that SEC13 siRNA did not always upregulate the level of an NMD target, whereas GNL2 siRNA did?

Referee #2:

Cáceres and colleagues use a genome-wide RNAi screen to identify novel NMD factor genes in C. elegans. NMD genes were selected by the criterion that their silencing restores GFP expression in worms expressing a GFP reporter containing a premature termination codon. The authors identify five putative NMD genes in nematodes that are all conserved. The authors go on to validate one of the five homologous genes as a NMD factor in Drosophila (nompA) and two out of the five homologous genes as NMD factors in human HeLa cells (GNL2 and SEC13). The authors establish an interaction between the human GNL2 and SEC13 proteins with the central NMD factor, UPF1, and provide some evidence that these factors participate in a regulatory feed-back loop.
The manuscript is very straightforward and the findings are highly significant. However, the work must be strengthened by providing additional evidence that the candidate NMD factors are actually functioning in the NMD pathway, as described below.
1. mRNA half-life analysis must be provided to authenticate that these are NMD factors. This is essential, as the hallmark of NMD is that it destabilizes mRNA.
2. For the putative mammalian NMD factors, a secondary assay (such as qPCR analysis of endogenous NMD substrates) should be performed to confirm that they are NMD factors.
3. The authors should explore, at least to some extent, indirect effects arising from knockdown of their putative NMD genes in mammalian cells. For example, the SMD RNA decay pathway is known to compete with NMD. If the putative factors identified inhibit SMD, their knockdown may promote SMD and subsequently inhibit NMD. Alternatively, the factors may be affecting translation. Because NMD requires translation, knockdown of the factors may inhibit translation and indirectly inhibit NMD. The authors should perform additional experimentation to rule out these possibilities.

Referee #3:

In this brief report, Casadio and colleagues show their results of a genome-wide RNAi screen for new NMD factors conducted in C. elegans. The Caceres lab has performed previously a very similar screen during which they discovered the two new NMD factors smgl1 and smgl. Compared to their previous screen, they use here an improved siRNA library that targets roughly 55% of the C. elegans genes. To identify NMD factors, they used the C. elegans strain PTCxi that expresses a GFP-based NMD reporter gene and searched for green worms. This screen identified 5 new genes that not only showed green fluorescence comparable to the positive control smg-2 but also accordingly increased mRNA levels in validation experiments. Unlike smg-2, but similar to the previously identified smgl-1 and smgl-2, these 5 new factors all showed developmental defects, suggesting that they may have additional functions in addition to NMD. Four of them have mammalian homologs (ngp-1 = GNL2, npp-20 = SEC13, aex-6 = RAB27A/B, and pbs-2 = PSMB7/10) and the fifths (noah-2) has a homolog in Drosophila (nompA). The domain compositions and alignments of the respective

homologs are documented in supplementary material.
The authors then went on to show that the nompA gene is also required for NMD in type I sense organs of the peripheral nervous system in Drosophila embryos, where this gene is expressed. This was shown using a GFP-based NMD reporter similar to the one used in the worms. To test if the four human homologs are also involved in NMD, they knocked down GNL2, SEC13, RAB27A, RAB27B and PSMB10 in HeLa cells expressing either a WT or a PTC-containing beta-globin reporter gene. GNL2 and SEC13 knockdown elevated PTC+ beta-globin mRNA, indicating that these two factors are also required for NMD in human cells. Knockdown of the other three factors did not lead to a significant increase of the NMD reporter mRNA, but a negative result in a knockdown experiment is of course not conclusive (see comment below). For SEC13 and GNL2, the authors showed that they co-immunoprecipitate with UPF1 in an RNase-resistant manner, suggesting that they interact with UPF1 via protein-protein interactions.

The paper is succinctly written and the data is clear and compelling. I have only two points that I find worthwhile addressing, both regarding Fig. 4:

1.) The authors should also try double knockdowns of RAB27A and RAB27B to test whether maybe these two proteins can substitute each other in NMD. Likewise, it is unclear why no knockdown of PSMB7 was done. I suggest to also knockdown this factor, alone and in combination with PSMB10.

2.) It is surprising that the knockdown of SEC13 has no effect on SMG1 mRNA (Fig 4E), given that the SMG1 transcript is a well-characterized NMD target. This should be discussed, or at least pointed out, in the manuscript.

1st Revision - authors' response 13 October 2014

Point-by point response to reviewers

Referee #1:

The authors use the same screen but a different C. elegans RNAi library than previously used to identify new NMD factors. This library includes dsRNAs that downregulate 1,736 genes not downregulated in the previous library and targets ~ 55% of the genome. The authors found and confirmed the existence of five new NMD factors:
C. elegans NGP-1 = human GNL2, a putative GTPase - yeast Nop2p, involved in ribosome biogenesis, processing and possibly nuclear export of pre-60 ribosomal particles.
C. elegans NNP-20 = human SEC13 and is constituent of the ER and NPC.
C. elegans AEX-6 involved in defecation = human RAB27A and B, member of the Rab smll GTPases
C. elegans PBS-2 is a member of the proteosome B-type family = human PSMB7 and 10

C. elegans NOAH-2 is the only of the five not conserved and has been shown to be required for embryonic and larval development.

The authors show that worms defective in NGP-1 are largely arrested in L1-L2 stage; worms defective in in NPP-20 are largely arrested in L2-L3; defects in AEX-6 progress with only a mild NPP-20-like defect; worms defective in in PBS-2 are arrested in L2; and in NOAH-2 in L2-L3.

The Drosophila ortholog of NOAH-2, NOMPA, which is expressed in the embryonic peripheral nervous system, was shown to function in NMD. So were GLN2 and SEC13 in human cells using NMD reporters.

This submission certainly adds to the field. It is a bit of a mish-mash. Some

explanation for why different species were used to assay for roles of the newly discovered C. elegans NMD factors is warranted. Additional comments are below.

This new genome-wide RNAi screen was performed in *C. elegans* and builds on the success of our previous screen that resulted in the identification of two novel NMD factors that were also shown to be involved in the NMD pathway in mammalian cells (DHX34 and NBAS, see Longman et al. (2007) *Genes Dev/* PubMed PMID: 17437990). The main difference here was the use of a different RNAi library that includes dsRNAs against 1,736 genes that were not targeted with the previously used RNAi library. We used the *C. elegans* experimental system for several reasons: i) this allowed us to compare this new screen with the old one and determine whether there were additional NMD factors in nematodes; ii) Since NMD is not an essential process in nematodes, the fact that the five novel genes identified here are essential for viability, strongly suggests that these novel NMD factors have additional functions, other than NMD, in *C. elegans*; iii) Due to the high degree of evolutionary conservation, we still could analyze the role of these newly identified NMD factors in mammalian cells and for this we chose HeLa cells as our experimental system. The only exception was the *noah-2* gene that does not have a human counterpart. For this, we studied its functional homolog in *Drosophila* and found that it acts in the NMD pathway in insects.

Comments
Page 9. Why wasn't C. elegans used to study NOAH-2 function in NMD prior to larval arrest.

The NMD function of NOAH-2 was studied at the point of visible GFP upregulation, that coincides with the appearance of a visible phenotype at day 3 of RNAi treatment. The same timescale was applied to all other genes.

Figure 4 legend. "or depleted of UPF1....PSMB7 and PSMB10 using a pool of four siRNAs". What was done was probably not what is written since each protein was probably downregulated using a pool of siRNAs. Also, the use of pools of siRNAs raises a concern for off-target effects.

There was a mistake in the Figure legend. It should read “depleted of UPF2”. This has been corrected now. Also, in all experiments described in this paper we used a pool of siRNAs that are specifically designed to reduce off-target effects (Dharmacon ON-TARGETplus siRNA).

Figure 4 legend. What were HBB mRNAs from the wild-type and NS39 reporters normalized to so that they could be compared? Also, what were the levels of endogenous NMD targets normalized to?

To normalize the levels of HBB mRNAs either in a wild-type or a NS39 version, we used ACTB and POLR2J as reference genes. We selected these reference genes because their mRNA levels are not regulated by NMD (based on microarray and qPCR data, see Longman et al. (2013) *Nucleic Acids Res*/PubMed PMID: 23828042)). We have also used the same reference genes to test the NMD response of endogenous NMD substrates (**see new Fig 4E**).

Pages 10-11. "co-immunoprecipitated" would be a better term than "interacted".

We agree with this and have changed the text accordingly.

Figure 4. Using tagged proteins is fine but their level relative to endogenous protein should be approximately the same. Was this true for Flag-tagged UPF1, T7-tagged GLN2 or HA-tagged SEC13?

The reviewer is right and we have tried to manipulate the levels of expression of tagged proteins relative to endogenous proteins. We have now succeeded in achieving levels of very moderate expression for Flag-UPF1 and T7-GNL2 and under those conditions, these proteins still interact (**see new Fig E4).** In the case of HA-SEC13, this has been more problematic; therefore, we decided not to include this experiment in the manuscript, since we can only detect the interaction under conditions of high overexpression for both proteins. It should be noted that the Izaurralde lab observed an interaction of another NMD factor, SMG7 with SEC13, (Loh B et al. (2013) Genes Dev/ PubMed PMID: 24115769).

We believe that this does not affect the main conclusions of this paper, since we have several independent lines of evidence to demonstrate a role for both GNL2 and SEC13 in the NMD response in human cells. These include **i)** upregulation of an NMD reporter following their individual depletion (**Fig 4A, B**) ii) half-life experiments showing that depletion of GNL2 and SEC13 affects the degradation rate of an NMD reporter (**New Fig 4C, D**) iii) A new experiment showing that depletion of GNL2 or SEC13 leads to upregulation of endogenous NMD substrates (**New Fig 4E**). The interaction with UPF1 is suggestive of a role in NMD, but it is still possible that many factors that act in the NMD pathway do not interact with UPF1. This will be the basis of future mechanistic studies.

Figure 4 legend. Please define "EV" as empty vector so there is no confusion.

Done

Figure 4C. What happened to the WB using anti-Flag? There is no band in the IP - possibly a lighter exposure is required.

This Figure was replaced with new Figure E4.

Figure 4E. The key should include "siRNA" since that is what were used. And, the x axis should include "mRNA".

This has been corrected and is now shown in Fig 4F.

Page 11. Last paragraph. How to the authors explain that SEC13 siRNA did not always upregulate the level of an NMD target, whereas GNL2 siRNA did?

It should be noted that not all NMD factors regulate the level of all mRNAs encoding NMD factors.
Therefore, it is not entirely surprising that GNL2 and SEC13 regulate a different subset of NMD transcripts. Importantly, both GNL2 and SEC13 regulate the levels of UPF1 mRNA.

© European Molecular Biology Organization

Referee #2:

Cáceres and colleagues use a genome-wide RNAi screen to identify novel NMD factor genes in C. elegans. NMD genes were selected by the criterion that their silencing restores GFP expression in worms expressing a GFP reporter containing a premature termination codon. The authors identify five putative NMD genes in nematodes that are all conserved. The authors go on to validate one of the five homologous genes as a NMD factor in Drosophila (nompA) and two out of the five homologous genes as NMD factors in human HeLa cells (GNL2 and SEC13). The authors establish an interaction between the human GNL2 and SEC13 proteins with the central NMD factor, UPF1, and provide some evidence that these factors participate in a regulatory feed-back loop.

The manuscript is very straightforward and the findings are highly significant. However, the work must be strengthened by providing additional evidence that the candidate NMD factors are actually functioning in the NMD pathway, as described below.

1. mRNA half-life analysis must be provided to authenticate that these are NMD factors. This is essential, as the hallmark of NMD is that it destabilizes mRNA.

This reviewer makes a very good suggestion that we have taken on board. We are now including new data showing that the half-life of an NMD-sensitive mRNA reporter is substantially increased upon siRNA-mediated depletion of GNL2 or SEC13 (**see new Fig 4C and 4D**). We are also showing that the half-life of an endogenous NMD target, ARHGEF18, is significantly increased upon depletion of GNL2 and SEC13 (**Fig E3D**).

2. For the putative mammalian NMD factors, a secondary assay (such as qPCR analysis of endogenous NMD substrates) should be performed to confirm that they are NMD factors.

As suggested, we are now including new data that shows that both GNL2 and SEC13 do indeed regulate the expression of endogenous NMD substrates **(new Fig 4E**). The endogenous NMD substrates were chosen based on our previous Microarray data that showed that they are responsive to depletion of different NMD factors (UPF1, DHX34, NBAS; Longman et al. (2013) *Nucleic Acids Res*/PubMed PMID: 23828042) and on published UPF1 targets (Mendell *et al.*, (2004) *Nat Genet*/PubMed PMID: 15448691). We are also showing that GNL2 and SEC13 regulate the half-life of an endogenous NMD substrate ARHGEF18 mRNA (**new Fig E3D).**

3. The authors should explore, at least to some extent, indirect effects arising from knockdown of their putative NMD genes in mammalian cells. For example, the SMD RNA decay pathway is known to compete with NMD. If the putative factors identified inhibit SMD, their knockdown may promote SMD and subsequently inhibit NMD. Alternatively, the factors may be affecting translation. Because NMD requires translation, knockdown of the factors may inhibit translation and indirectly inhibit NMD. The authors should perform additional experimentation to rule out these possibilities.

This is an interesting idea. We have carried out experiments that revealed that depletion of GNL2 or SEC13 affects the mRNA levels of a subset of SMD targets (selected from Gong *et al.* (2009) *Genes Dev*/PubMed PMID: 19095803). However, the results were not conclusive, since knockdown of GNL2 or SEC13 resulted in cases of both upregulation or downregulation of SMD

© European Molecular Biology Organization

targets. Thus, although we cannot rule this out completely, it is unlikely that GNL2 or SEC13 regulate mRNA level of their targets via modulating SMD response. These data can be provided, if requested.

As for the translation hypothesis, the very nature of the RNAi screen in *C. elegans* requires that the GFP-lacZ reporter is indeed translated. Thus, the upregulation of GFP expression upon depletion of individual NMD factors cannot be explained by a general inhibitory effect on translation during the screen. Nonetheless, to confirm that this is the case in mammalian cells, we are now including new data that shows that depletion of GNL2 or SEC13 does not result in a general inhibition of translation (**new Fig E3 E and F**).

Referee #3:

In this brief report, Casadio and colleagues show their results of a genome-wide RNAi screen for new NMD factors conducted in C. elegans. The Caceres lab has performed previously a very similar screen during which they discovered the two new NMD factors smgl1 and smgl. Compared to their previous screen, they use here an improved siRNA library that targets roughly 55% of the C. elegans genes. To identify NMD factors, they used the C. elegans strain PTCxi that expresses a GFP-based NMD reporter gene and searched for green worms. This screen identified 5 new genes that not only showed green fluorescence comparable to the positive control smg-2 but also accordingly increased mRNA levels in validation experiments. Unlike smg-2, but similar to the previously identified smgl-1 and smgl-2, these 5 new factors all showed developmental defects, suggesting that they may have additional functions in addition to NMD. Four of them have mammalian homologs (ngp-1 = GNL2, npp-20 = SEC13, aex-6 = RAB27A/B, and pbs-2 = PSMB7/10) and the fifths (noah-2) has a homolog in Drosophila (nompA). The domain compositions and alignments of the respective homologs are documented in supplementary material.
The authors then went on to show that the nompA gene is also required for NMD in type I sense organs of the peripheral nervous system in Drosophila embryos, where this gene is expressed. This was shown using a GFP-based NMD reporter similar to the one used in the worms. To test if the four human homologs are also involved in NMD, they knocked down GNL2, SEC13, RAB27A, RAB27B and PSMB10 in HeLa cells expressing either a WT or a PTC-containing beta-globin reporter gene. GNL2 and SEC13 knockdown elevated PTC+ beta-globin mRNA, indicating that these two factors are also required for NMD in human cells. Knockdown of the other three factors did not lead to a significant increase of the NMD reporter mRNA, but a negative result in a knockdown experiment is of course not conclusive (see comment below). For SEC13 and GNL2, the authors showed that they co-immunoprecipitate with UPF1 in an RNase-resistant manner, suggesting that they interact with UPF1 via protein-protein interactions.

The paper is succinctly written and the data is clear and compelling. I have only two points that I find worthwhile addressing, both regarding Fig. 4:

1.) The authors should also try double knockdowns of RAB27A and RAB27B to test whether maybe these two proteins can substitute each other in NMD.

We have followed this suggestion and are now including double knock-downs for RAB27A and RAB27B that show that even the combined depletion of these factors does not result in an increase of the human β-globin (HBB) NMD reporter gene (**New Fig 4A and 4B**).

Likewise, it is unclear why no knockdown of PSMB7 was done. I suggest to also knockdown this factor, alone and in combination with PSMB10.

As for PSMB7, its depletion is lethal at day 5. We are now including data showing the effect of PSMB7 and PSMB10 knockdown, individually and in combination, at day 4. These data revealed unspecific effects on both the wt and the NMD HBB reporters (**new Fig E3A, B**).

2.) It is surprising that the knockdown of SEC13 has no effect on SMG1 mRNA (Fig 4E), given that the SMG1 transcript is a well-characterized NMD target. This should be discussed, or at least pointed out, in the manuscript.

Reviewer 1 raised a similar point. We are not sure why SMG1 mRNA levels are not regulated by SEC13. It should be noted that not all NMD factors regulate the level of all mRNAs encoding NMD factors. Therefore, it is not entirely surprising that GNL2 and SEC13 regulate a different subset of NMD transcripts. Importantly, both GNL2 and SEC13 regulate the level of UPF1 mRNA.

2nd Editorial Decision 23 October 2014

We have received now the three referee reports on your revised manuscript and as you will see, all referees support publication of the study in our journal.

However, all referees also have minor suggestions for how the manuscript and data presentation could be improved, which should all be addressed and incorporated in the final version of the manuscript before we can proceed with the official acceptance.

I look forward to seeing a final version of your manuscript as soon as possible.

REFEREE REPORTS:

Referee #1:

The responses to my queries are fine, and in my view the manuscript is acceptable for publication. I wish the authors had incorporated why they used different species into the paper rather than only in their response to reviewers. I wonder if readers will also wonder this about their approach. However, aside from this, the manuscript is very nice.

Referee #2:

This revised manuscript is much improved. A modest concern is that the RNA half-life experiment (Fig. 4C & D) has rather scattered data points, but the trend is clear and thus it is acceptable. The authors offered to provide the SMD results requested by Reviewer 2 (point 3), which I strongly suggest should be included in the final publication, even though both up- and down-regulation was observed. Overall, this is an outstanding study that brings forth new factors that function in a highly conserved pathway that is of increasing interest to both the basic science and medical communities.

Referee #3:

The two points that I raised in my initial evaluation have been addressed adequately.

Per request of another reviewer, the authors have now also performed half-life measurements to show that the observed changes in RNA levels are really due to altered mRNA stability. This is laudable and further strengthens the conclusion that the identified factors play a role in NMD, but the data (Fig. 4C and D) is displayed incorrectly. As the name "half-life" implies, the disappearance of the RNA is expected to follow an expontential decay curve and it is therefore not correct to calculate a linear regression line from the data points plotted on a linear y-axis. Instead, they should use a semi-logarithmic plot (y-axis log scale). Now they can determine the regression line and obtain from its slope the half-life of the mRNA. It is very obvious for NS39 b-globin Mock that the indicated half-life of 5 h is wrong, the plot clearly shows that it is around 2 h.

Although EMBO Reports in general does not allow two rounds of revision, I strongly suggest that the authors should be allowed to correct this dilettantish mistake before publication.

2nd Revision - authors' response 30 October 2014

Point-by point response to reviewers

Referee #1:

The responses to my queries are fine, and in my view the manuscript is acceptable for publication. I wish the authors had incorporated why they used different species into the paper rather than only in their response to reviewers. I wonder if readers will also wonder this about their approach. However, aside from this, the manuscript is very nice.

We are now following this suggestion and have incorporated one paragraph referring to the use of different species on Pages 10-11.

Referee #2:

This revised manuscript is much improved. A modest concern is that the RNA half-life experiment (Fig. 4C & D) has rather scattered data points, but the trend is clear and thus it is acceptable. The authors offered to provide the SMD results requested by Reviewer 2 (point 3), which I strongly suggest should be included in the final publication, even though both up- and down-regulation was observed. Overall, this is an outstanding study that brings forth new factors that function in a highly conserved pathway that is of increasing interest to both the basic science and medical communities.

We respectfully disagree with this Referee on this aspect and think that the SMD experiments that we carried out were indeed too preliminary and are not entirely conclusive. Therefore, we would prefer not to include these results in the paper.

Referee #3:

The two points that I raised in my initial evaluation have been addressed adequately.

Per request of another reviewer, the authors have now also performed half-life measurements to show that the observed changes in RNA levels are really due to altered mRNA stability. This is laudable and further strengthens the

© European Molecular Biology Organization

conclusion that the identified factors play a role in NMD, but the data (Fig. 4C and D) is displayed incorrectly. As the name "half-life" implies, the disappearance of the RNA is expected to follow an expontential decay curve and it is therefore not correct to calculate a linear regression line from the data points plotted on a linear y-axis. Instead, they should use a semi-logarithmic plot (y-axis log scale). Now they can determine the regression line and obtain from its slope the half-life of the mRNA. It is very obvious for NS39 b-globin Mock that the indicated half-life of 5 h is wrong, the plot clearly shows that it is around 2 h.

Although EMBO Reports in general does not allow two rounds of revision, I strongly suggest that the authors should be allowed to correct this dilettantish mistake before publication.

We apologise for this mistake and have now corrected this, as suggested by this reviewer, by fitting an exponential decay curve and displaying the data using a semi-logarithmic plot (y-axis log2 scale). This resulted in changes to Figures 4C, D and to supplementary Figure E3D.

3rd Editorial Decision 03 November 2014

I am very pleased to accept your manuscript for publication in the next available issue of EMBO reports.

Thank you for your contribution to EMBO reports and congratulations on a successful publication. Please consider us again in the future for your most exciting work.

© European Molecular Biology Organization
